# Sustainable Design and DoE-Based Optimization of Polymeric Systems for FDM 3D-Printed Indomethacin Amorphous Solid Dispersions

**DOI:** 10.3390/ph19040562

**Published:** 2026-04-01

**Authors:** Ioannis Pantazos, Christos Cholevas, Christos Vlachokostas, Afroditi Kapourani, Panagiotis Barmpalexis

**Affiliations:** 1 Laboratory of Pharmaceutical Technology, Division of Pharmaceutical Technology, School of Pharmacy, Faculty of Health Sciences, Aristotle University of Thessaloniki, 541 24 Thessaloniki, Greece; ipantazo@pharm.auth.gr (I.P.); ccholevas@pharm.auth.gr (C.C.); pbarmp@pharm.auth.gr (P.B.); 2 Sustainability Engineering Laboratory, Department of Mechanical Engineering, Aristotle University of Thessaloniki, 541 24 Thessaloniki, Greece; vlahoco@meng.auth.gr; 3 Natural Products Research Centre of Excellence-AUTH (NatPro-AUTH), Center for Interdisciplinary Research and Innovation (CIRI-AUTH), 570 01 Thessaloniki, Greece

**Keywords:** amorphous solid dispersions, fused deposition modeling, hot-melt extrusion, sustainability-by-design, electrical energy optimization, design of experiments

## Abstract

**Background/Objectives**: Amorphous solid dispersions (ASDs) produced via hot-melt extrusion (HME) and fused deposition modeling (FDM) 3D printing represent a promising strategy for improving the performance of poorly water-soluble drugs. However, the integrated HME-FDM workflow is inherently energy-intensive, and sustainability considerations are rarely incorporated into formulation and process optimization. The present study aimed to develop and optimize indomethacin (IND) ASDs using a systematic Design of Experiment (DoE) framework that integrates electrical energy consumption as a quantitative response alongside pharmaceutical performance attributes. **Methods**: Polymer–plasticizer miscibility was screened using hot-stage microscopy, followed by filament preparation via HME. A factorial DoE was applied to optimize drug loading and extrusion temperature considering electrical energy consumption, extrusion yield, encapsulation efficiency, and residual crystallinity. Solid-state characterization was performed using DSC and XRD. The optimized filament was subsequently subjected to geometry screening and a second DoE to optimize platform temperature, nozzle temperature, and printing speed with respect to printing time, electrical energy consumption, and drug assay. **Results**: Complete drug amorphization was achieved within a defined thermal window, with residual crystallinity governed by kinetic dissolution constraints at lower extrusion temperatures. Electrical energy demand during both HME and FDM was strongly influenced by thermal setpoints and process duration. Multi-response overlay analysis identified sustainability-oriented operating windows for both stages. Experimental validation confirmed close agreement between predicted and observed responses, demonstrating simultaneous reduction in electrical demand and maintenance of dose accuracy and solid-state stability. **Conclusions**: This study demonstrates that electrical energy consumption can be systematically embedded as a quantitative design variable in pharmaceutical process optimization. The proposed dual-stage DoE strategy establishes a rational framework for developing 3D-printed ASD dosage forms that balance molecular performance and environmental efficiency.

## 1. Introduction

Poor aqueous solubility remains one of the most significant challenges in contemporary pharmaceutical development, particularly for a wide range of active pharmaceutical ingredients (APIs) intended for oral administration [1,2]. Amorphous solid dispersions (ASDs) are widely recognized as one of the most effective formulation strategies to overcome this limitation, as they enable the molecular dispersion of poorly soluble drugs within polymeric carriers, thereby enhancing apparent solubility, dissolution rate, and oral bioavailability [3,4,5,6].

In recent years, three-dimensional (3D) printing, and particularly fused deposition modeling (FDM), has emerged as an innovative and highly promising platform for the development of ASD-based pharmaceutical dosage forms [7]. This approach enables precise control over dosage form geometry, internal architecture, and drug loading. FDM has attracted considerable interest due to its ability to fabricate patient-specific dosage forms that are difficult to achieve using conventional manufacturing techniques [8,9,10]. Key advantages include dose personalization, on-demand manufacturing, reduced batch sizes, and the production of complex solid dosage forms with high geometrical accuracy and reproducibility [11]. These features position FDM as a powerful enabling technology for next-generation ASD-based drug delivery systems.

Beyond its pharmaceutical advantages, FDM-based 3D printing is often perceived as a more environmentally favorable alternative to traditional manufacturing routes. Reduced material waste, limited post-processing requirements, and lower costs for producing geometrically complex designs have contributed to the view of FDM as a key technology for sustainable pharmaceutical production [12]. However, this perception warrants critical reassessment when the full manufacturing workflow is considered.

Despite its transformative potential, additive manufacturing presents environmental challenges that have frequently been underestimated or insufficiently explored. The sustainability of FDM 3D printing is strongly influenced by the environmental profile of the raw materials employed, as well as by the substantial energy demands associated with printer operation [13,14]. As a fully digital manufacturing approach, 3D printing relies heavily on continuous electricity consumption, which constitutes a major contributor to carbon dioxide emissions, particularly when energy is derived from non-renewable sources. Furthermore, FDM-based pharmaceutical manufacturing requires the prior preparation of drug-loaded polymeric filaments (FILs), most commonly produced via hot-melt extrusion (HME) [15]. This introduces a two-step, high-temperature workflow (HME followed by FDM). If not carefully optimized, this process may conflict with sustainability objectives due to cumulative energy demand.

These challenges must be viewed within the broader industrial context shaped by the increasing recognition of climate change as an urgent global challenge. Environmental mitigation and long-term sustainability now play a dominant role in shaping strategic priorities across diverse industrial sectors [16]. The systematic adoption of environmentally responsible practices, such as energy efficiency optimization, waste minimization and valorization, and alignment with circular economy principles, is driving a transition from the paradigm of *Industry 4.0* toward the more human- and sustainability-centered concept of *Industry 5.0* [17,18,19]. While sectors such as transportation, manufacturing, and mining have already implemented substantial measures to reduce their environmental footprint through energy optimization and digitalization, sustainability considerations have received comparatively limited attention within the pharmaceutical industry [20]. As a result, pharmaceutical manufacturing is widely regarded as an energy-intensive sector, with reported CO_2_ emissions approximately 55% higher than those of the automotive industry [21].

Within this framework, there is an urgent need for the rational optimization of 3D-printed pharmaceutical drug delivery systems, not only in terms of product performance but also with respect to environmental sustainability. This necessity extends to the careful selection and optimization of the used excipients, as well as to the systematic control of critical process parameters associated with both HME and FDM-based 3D printing. Rather than relying on empirical trial-and-error approaches, structured and data-driven optimization strategies are required to balance printability, drug stability, dosage form performance, and energy efficiency. Previous work [13] has demonstrated that energy consumption can be quantitatively measured across different 3D printing platforms and operational settings and may serve as a proxy for environmental impact, thereby providing a framework for sustainability assessment in pharmaceutical manufacturing. In parallel, studies within the broader additive manufacturing field have examined the influence of processing parameters on energy consumption and process efficiency, primarily from an engineering optimization perspective [22]. However, these approaches remain largely descriptive and are decoupled from formulation development, as energy consumption is evaluated independently of drug–polymer interactions, solid-state behavior, and critical quality attributes of the final dosage form. Consequently, the interplay between molecular-level phenomena governing ASDs formation and process-level energy demand remains insufficiently understood.

In this context, the present study introduces a formulation process integrated framework in which electrical energy consumption is explicitly incorporated as a quantitative response variable within a dual-stage Design of Experiment (DoE) strategy spanning both HME and FDM. Indomethacin (IND) was selected as a model API for ASD development, as it represents one of the most extensively investigated compounds in ASD research due to its poor aqueous solubility [23,24,25]. Process and formulation optimization encompassed both the HME and FDM stages, including the rational selection of polymeric carriers and plasticizers to ensure printability, drug stabilization, and dosage form performance. Specifically, polymeric carriers were selected and evaluated from polyvinyl alcohol (PVA) and Soluplus^®^ (SOL), while various plasticizers—mannitol (MAN), triethyl citrate (TEC), citric acid (CA), and polyethylene glycol (PEG)—were evaluated for their ability to modulate glass transition temperature (Tg), reduce melt viscosity, and enhance processability [26,27,28]. In parallel, the environmental impact of the integrated HME-FDM manufacturing workflow was systematically assessed, with particular emphasis on energy consumption as a key sustainability indicator. The selection of electrical energy consumption as a process metric is consistent with prior studies in pharmaceutical and additive manufacturing systems, where it has been employed as a quantifiable proxy for environmental impact [13,22]. Specifically, electrical energy demand represents a proxy metric that can be translated into carbon emissions using established conversion factors (e.g., CO_2_eq per kWh), although such conversion is inherently dependent on the electricity generation mix and system boundaries. For example, according to European Environment Agency data, the greenhouse gas emission intensity of electricity generation in Greece is approximately 0.25–0.35 kg CO_2_/kWh [29]. The proposed framework therefore establishes a direct connection between material properties, processing conditions, and energy consumption, enabling identification of operating windows that balance solid-state performance with reduced resource intensity. To the best of our knowledge, this represents the first systematic attempt to integrate energy consumption across the full HME-FDM workflow for the development of ASDs within a unified DoE framework. By coupling DoE-driven formulation and process optimization with energy-based environmental evaluation, this study focuses to the development of advanced 3D-printed ASD-based pharmaceutical dosage forms [30].

## 2. Results

### 2.1. Thermal Stability Studies

Thermal stability is a key factor for the rational design of ASDs manufactured via melt-based technologies, as thermal degradation may compromise drug potency, alter polymer functionality, and negatively affect the printability and performance of the final dosage forms. TGA was performed to evaluate the thermal stability of the investigated raw materials and to determine their suitability for melt-based processing during HME and FDM.

**Figure 1:**
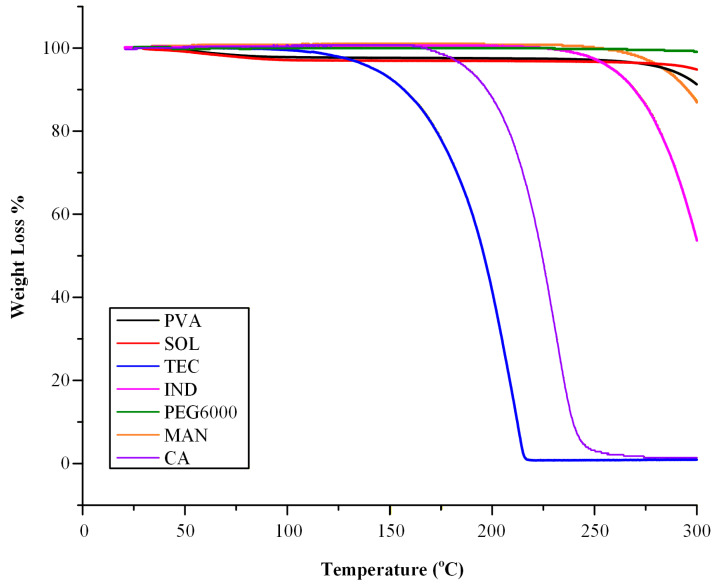
TGA thermograms of the investigated raw materials.

The TGA thermogram of pure PVA demonstrated that the polymer remained thermally stable up to approximately 250 °C (Figure 1). The initial weight loss observed below 100 °C, corresponding to approximately 5%, was attributed to the evaporation of absorbed moisture during storage, reflecting the hygroscopic nature of the polymer. Similarly, the TGA profile of SOL indicated that the polymer remained thermally stable up to approximately 275 °C. A minor weight loss of 4.2% recorded below 100 °C was also attributed to the loss of absorbed moisture. Beyond this temperature range, no significant mass loss was observed within the typical processing window for HME and FDM, confirming its suitability for melt-based processing.

The thermogram of MAN (same figure) revealed that the excipient remained thermally stable without signs of degradation up to approximately 250 °C. PEG6000 also demonstrated excellent thermal stability, with no detectable thermal degradation up to 300 °C, indicating a wide processing window for its potential use as a plasticizer in melt-processed systems. In contrast, CA and TEC demonstrated limited thermal stability, exhibiting significant mass loss and evidence of thermal degradation at temperatures exceeding approximately 170 °C and 130 °C, respectively. These degradation thresholds fall within or below the thermal range required for processing IND, whose melting point is approximately 160 °C [31]. Consequently, both plasticizers may be susceptible to thermal degradation during FIL preparation by HME. Their restricted thermal stability therefore represents a potential limiting factor for melt-based processing, particularly under conditions requiring elevated temperatures to ensure complete drug amorphization and adequate melt flow. Finally, the TGA thermogram of pure IND demonstrated that the drug remained thermally stable up to approximately 240 °C. These findings indicate that extrusion and printing processes conducted below this temperature threshold are unlikely to induce significant thermal degradation of the drug substance.

### 2.2. Miscibility Studies

The miscibility between candidate polymeric carriers and plasticizers was systematically investigated to identify suitable systems for the development of IND-ASDs intended for HME and FDM 3D printing. The components’ miscibility during ASD preparation is closely related to the physical stability of the system [32,33], as immiscibility may lead to phase separation and increased recrystallization tendency of the API [34]. However, reliable prediction of drug–polymer miscibility remains challenging, and no single method can provide a definitive assessment [32]. Although differential scanning calorimetry (DSC) and the identification of a single Tg are commonly employed, this approach presents limitations in multicomponent systems due to overlapping transitions and reduced sensitivity [35]. In this context, hot-stage microscopy (HSM) was employed as a practical and process-relevant tool to evaluate melt-state behavior under conditions relevant to HME. HSM enables direct visualization of phase behavior during heating, allowing identification of phase separation or crystalline remnants. Within the scope of the present study, HSM was considered sufficient as a preliminary screening method to assess melt-state compatibility, which is a critical prerequisite for successful ASD formation via HME.

**Figure 2:**
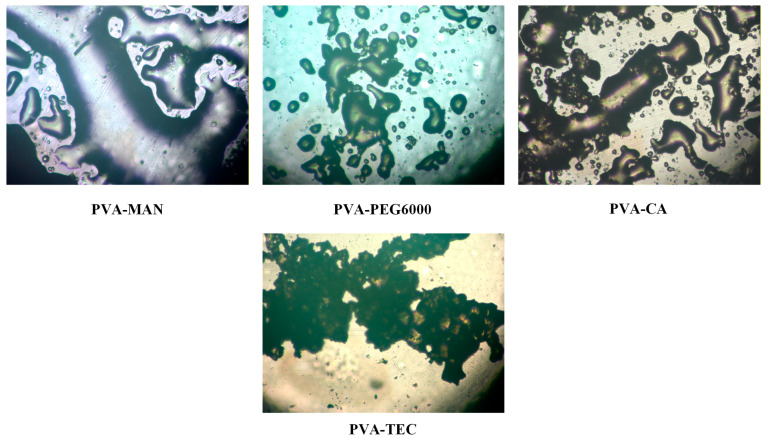
HSM images of molten PVA systems with the investigated plasticizers.

**Figure 3:**
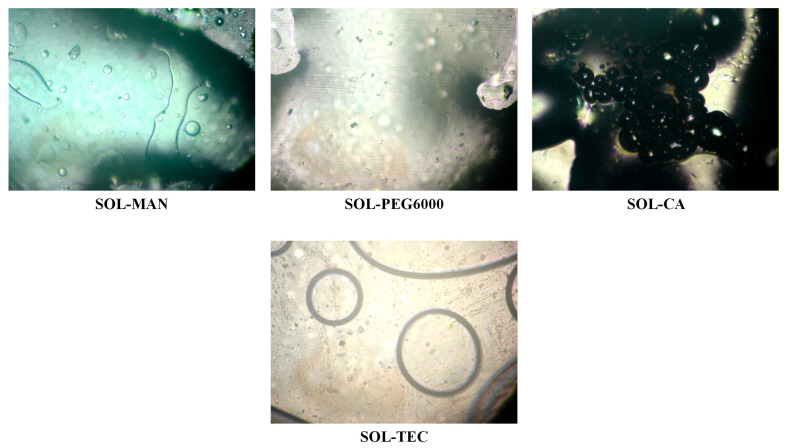
HSM images of molten SOL systems with the investigated plasticizers.

Initially, the miscibility of PVA and SOL with the selected plasticizers (i.e., MAN, PEG6000, CA, and TEC) was evaluated in the molten state using HSM. Representative photographs are presented in Figure 2 and Figure 3.

As shown in Figure 2, PVA exhibited satisfactory miscibility with both MAN and PEG 6000, as indicated by the formation of homogeneous molten phases without visible phase separation. In contrast, in the PVA-CA and PVA-TEC systems, thermal degradation of the plasticizers was observed at temperatures approaching the melting point of PVA, rendering these combinations unsuitable for further development under the examined processing conditions.

The miscibility behavior of SOL differed depending on the plasticizer employed (Figure 3). The SOL-MAN and SOL-CA systems displayed clear phase separation during melting, forming distinct molten regions and indicating lack of miscibility. Conversely, both SOL-PEG6000 and SOL-TEC systems formed uniform molten phases without visible separation, suggesting favorable miscibility and thermal compatibility.

Based on these findings, four polymer–plasticizer systems (i.e., PVA-MAN, PVA-PEG 6000, SOL-PEG6000, and SOL-TEC) were further evaluated in the presence of IND to assess their suitability for ASD formation.

**Figure 4:**
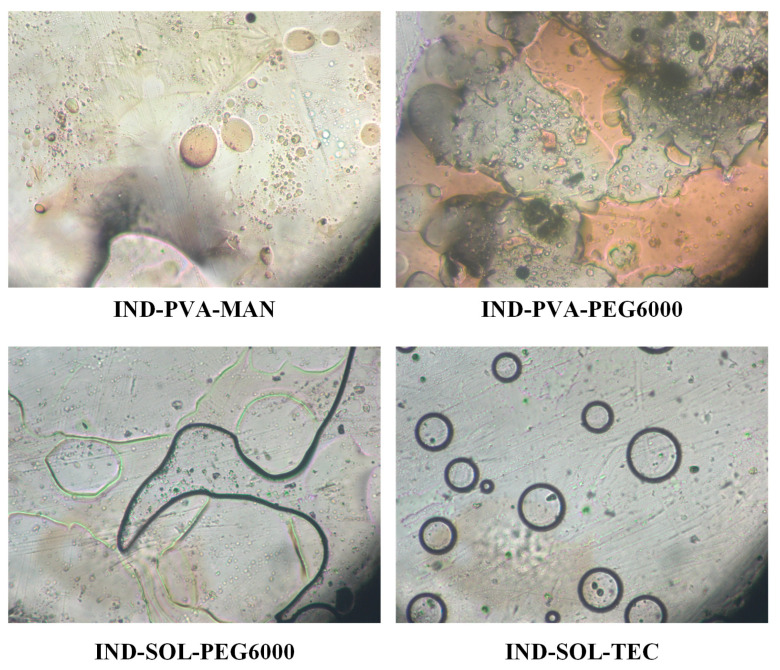
HSM images of the investigated polymer–plasticizer systems in the presence of the IND.

The incorporation of IND significantly affected system miscibility. As illustrated in Figure 4, all PVA-based ternary systems exhibited pronounced phase separation upon melting, regardless of the plasticizer used. Distinct molten domains corresponding to individual components were observed, indicating inadequate miscibility and limited potential for homogeneous ASD formation. Consequently, IND-PVA-MAN and IND-PVA-PEG6000 systems were excluded from further investigation.

For SOL-based systems, different behaviors were observed. The IND-SOL-PEG6000 system exhibited phase separation, indicating poor miscibility among the components. In contrast, the IND-SOL-TEC system formed a single homogeneous molten phase without visible separation, demonstrating satisfactory miscibility between IND, SOL, and TEC. Based on these results, SOL was selected as the polymeric carrier and TEC as the plasticizer for subsequent HME and FDM optimization studies.

### 2.3. Selection of Plasticizer Content

Following the identification of SOL and TEC as the most suitable polymer–plasticizer combination based on miscibility studies, preliminary HME experiments were conducted to determine the optimal TEC concentration required to produce FILs with appropriate handling properties.

Three formulations containing a fixed IND loading of 10% *w*/*w* and varying TEC concentrations (5, 10, and 15% *w*/*w*) were prepared and extruded under identical processing conditions. The investigated concentration range (5–15% *w*/*w*) was selected to systematically modulate the extent of polymer plasticization while remaining within the plasticizer levels commonly reported for printable pharmaceutical FILs.

Plasticizers such as TEC function by reducing intermolecular polymer–polymer interactions, increasing free volume, and lowering the glass transition temperature (Tg) of the matrix. This Tg depression reduces segmental relaxation time and melt viscosity, thereby decreasing extrusion torque and facilitating continuous strand formation. However, excessive reduction in Tg may compromise solid-state mechanical strength by increasing molecular mobility at ambient conditions [36,37]. Thus, plasticizer concentration must be optimized to achieve sufficient melt flow during HME while preserving FIL stiffness required for reliable feeding during FDM.

Evidence supporting this range is provided by Maru et al. [38], who evaluated the impact of TEC (and PEG 6000) on the thermal and rheological behavior of HME formulations and reported that TEC at 5–10% *w*/*w* enabled suitable processing by HME, demonstrating substantial plasticization efficiency at relatively low concentrations. The authors further demonstrated that increasing TEC loading progressively altered rheological behavior, with higher concentrations leading to marked viscosity reduction and changes in shear-thinning characteristics, confirming the strong plasticization efficiency of TEC in melt-processed systems. These findings support the investigation of TEC at relatively low levels as a starting point for achieving adequate melt processability without excessive softening of the polymeric matrix. In addition, a broad formulation survey by Pereira et al. [28] compiling HME-FDM FIL studies reported that the plasticizer content typically ranges between ~5% and 20% *w*/*w* across printable pharmaceutical formulations.

At this study, FILs prepared with 5% *w*/*w* TEC exhibited pronounced brittleness and fractured easily during handling, indicating insufficient plasticization of the polymeric matrix. Similarly, FILs containing 15% *w*/*w* TEC demonstrated increased fragility, likely due to over-plasticization of the system and reduced structural cohesion. In contrast, FILs produced with 10% *w*/*w* TEC displayed improved flexibility, and sufficient mechanical strength to withstand handling and further processing. Based on these observations, a TEC concentration of 10% *w*/*w* was selected as the optimal plasticizer content for subsequent formulation development, HME optimization, and FDM 3D printing studies. This concentration provided a balanced combination of flexibility and mechanical robustness, ensuring the production of FILs suitable for continuous feeding during 3D printing while maintaining formulation stability.

### 2.4. DoE-Driven Optimization of HME FILs: Performance–Sustainability Relationships

A DoE framework was applied to simultaneously optimize FIL manufacturability and key quality attributes while explicitly quantifying electrical energy consumption (Y_1_) as a sustainability-relevant response and FIL extrusion yield (Y_2_), IND’s encapsulation efficiency (Y_3_) and IND’s residual crystallinity (Y_4_) as drug formulation-relevant responses. The experimental design and corresponding response values obtained from the factorial DoE are summarized in Table 1. The investigated design space was defined by two critical process and formulation variables: IND loading (5–20% *w*/*w*) and extrusion temperature (110–150 °C). These factors were selected based on preliminary formulation screening and thermal stability considerations, with the aim of simultaneously optimizing FIL performance and reducing the environmental footprint associated with HME processing. The experimental results revealed a measurable variation across all responses, confirming the sensitivity of FIL properties and process sustainability metrics to both formulation composition and processing temperature.

Across the experimental runs, Y_1_ ranged from 0.144 to 0.188 KWh per extrusion run, demonstrating measurable differences in electrical demand within the studied formulation-process space. Electrical energy demand during HME arises from both thermal heating requirements and mechanical work associated with melt transport and shear. Recent quantitative assessments show that electricity demand can be a dominant driver of the CO_2_ footprint of pharmaceutical 3D printing, and that parameter optimization can meaningfully reduce energy consumption and associated emissions [21]. Therefore, minimizing Y_1_ provides a rational pathway to reduce climate impact during melt-based processing.

Electrical energy consumption is defined as W=V×I, where *V* is the applied voltage and *I* the current intensity. Therefore, variations in recorded current profiles directly reflect changes in mechanical resistance and melt rheology during processing. To evaluate energy consumption, the average voltage and current values were calculated for each triplicate extrusion under identical DoE conditions. The corresponding profiles are provided in the Supplementary Material (Figures S1 and S2). The differences observed in the total data recording duration across the electrical profiles correspond to the total time required for complete extrusion of the entire feed material from the extruder. In all experimental runs, the same mass of physical mixture (PM) was introduced as feed; however, the time required to complete extrusion varied depending on the processing conditions. These differences are attributed to variations in melt rheology, which influence mass transport through the screw and die, and consequently affect the overall extrusion rate. Therefore, the differing time scales presented in the electrical recording diagrams do not reflect differences in initial feed quantity but rather the processing time necessary under each specific formulation–temperature combination.

ANOVA indicated that the selected factorial model for Y_1_ was significant (*p* < 0.0001), with A (IND content), B (extrusion temperature), and AB interaction all statistically significant terms (Table 2). The model showed strong fit statistics (R^2^ ≈ 0.92; predicted R^2^ ≈ 0.82; adequate precision ≈ 16), and diagnostic plots supported model adequacy (no transformation required; acceptable residual behavior; no influential outliers). The coded model for Y_1_ (Y_1_ = 0.1624 − 0.0064*A + 0.0120*B − 0.0027*AB) indicated a negative coefficient for A and a positive coefficient for B, demonstrating that increasing drug loading decreased energy demand, whereas increasing extrusion temperature increased energy demand; the significant AB term confirmed that the effect of temperature depended on drug loading. At first glance, the positive temperature-energy correlation is intuitive: higher barrel temperatures require greater electrical heating power and may increase heat losses to the environment. However, the effect of drug loading on energy consumption requires deeper mechanistic interpretation.

IND, when incorporated at elevated concentrations, likely reduces the effective melt viscosity of the SOL-TEC matrix under the examined conditions. Although IND does not reach its crystalline melting point at 110 °C, it can dissolve into the polymeric melt, behaving as a low-molecular-weight component that perturbs polymer–polymer interactions and increases free volume [39]. In SOL-based systems, such effects have been associated with modifications in thermal behavior, including depression of the Tg, which is indicative of increased molecular mobility within the polymer matrix [40]. This Tg reduction is interpreted as a manifestation of plasticization and is associated with reduced melt resistance and enhanced chain mobility under processing conditions. Such drug-induced plasticization [41] reduces chain entanglement density and lowers shear resistance within the extruder. Because mechanical energy input during HME is proportional to torque and shear stress, reduced melt viscosity translates into lower mechanical work requirements [36]. In this context, the decrease in energy demand observed in the present study is consistent with a potential reduction in melt resistance during extrusion at higher drug loadings. However, it should be emphasized that this interpretation is based on indirect evidence derived from energy consumption trends and literature-reported thermal behavior and was not directly confirmed through rheological measurements. Therefore, the relationship between drug loading and melt viscosity should be considered a mechanistically supported hypothesis rather than a directly validated causal relationship.

The negative coefficient of drug loading in the Y_1_ model therefore suggests that IND contributes to viscosity modulation, decreasing mechanical energy demand. The significant interaction between temperature and drug loading further indicates that this plasticization effect becomes more pronounced at elevated temperatures, where diffusion and mixing efficiency increase.

Extrusion yield (Y_2_) varied considerably across the experimental domain, ranging from approximately 24% to 47%. ANOVA demonstrated that both IND loading (A) and extrusion temperature (B) significantly affected extrusion yield. Model coefficients (Y_2_ = 36.50 + 3.88*A − 6.02*B) indicated that increasing drug loading positively influenced Y_2_, whereas increasing extrusion temperature exerted a negative effect, likely due to increased material softening and adhesion phenomena at elevated temperatures that may compromise material recovery.

Encapsulation efficiency (Y_3_) ranged from values in the mid-80% region to slightly above 110% across the design space (Table 1). ANOVA identified IND loading (A), extrusion temperature (B), and their interaction (AB) as significant contributors to Y_3_ variability. Model coefficients (Y_3_ = 93.76 − 6.61*A + 5.86*B − 3.37*AB) indicated that increasing drug loading tended to decrease encapsulation efficiency, whereas increasing extrusion temperature improved drug incorporation within the polymeric matrix. This behavior is consistent with enhanced melt homogeneity and drug dispersion at elevated processing temperatures. Encapsulation values exceeding 100% were observed in selected runs, which may be attributed to analytical variability, sampling heterogeneity, or minor deviations in calibration and weighing accuracy at low sample masses. Importantly, the analytical method was applied consistently across all experimental runs, and the observed trends remained internally coherent with formulation and processing variables. Therefore, the data are considered suitable for comparative evaluation within the DoE framework, where relative differences between conditions are of primary relevance.

**Figure 5:**
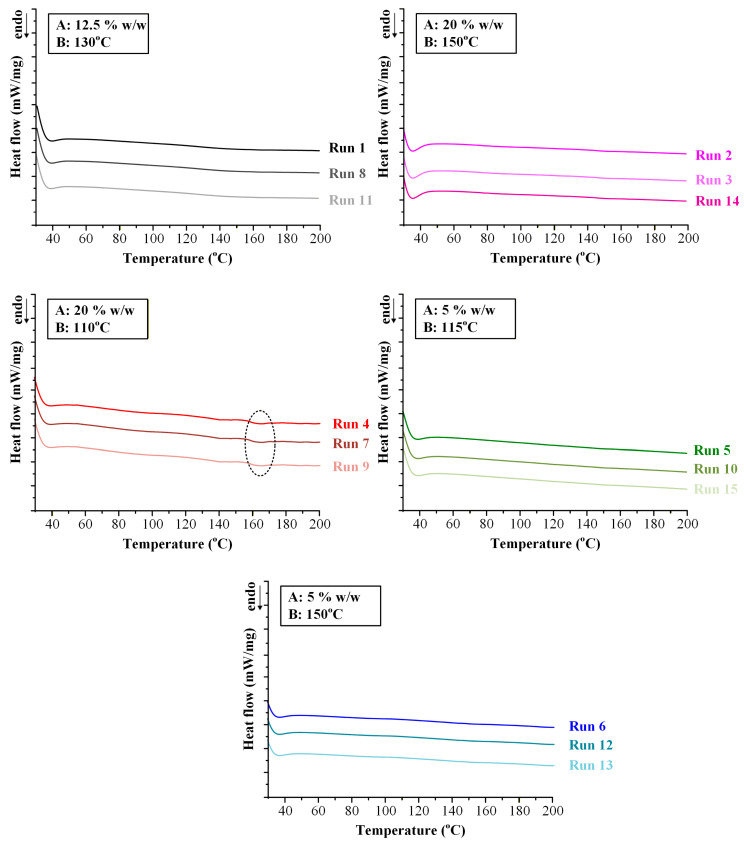
DSC thermograms of FIL correspond to the individual experimental runs of the DoE (run numbers indicated in the figures). Thermograms are grouped according to drug loading (A: 5%, 12.5%, and 20% *w*/*w* IND) and extrusion temperature (B: 110 °C, 130 °C, and 150 °C).

Residual crystallinity (Y_4_) was equal to zero in the majority of experimental runs, indicating successful amorphization of IND during the HME process, as supported by DSC thermograms (Figure 5). Non-zero crystallinity values (~7.5%) were observed only under conditions of high drug loading (20% *w*/*w*) combined with low extrusion temperature (110 °C).

Mechanistically, this response is governed by kinetic constraints rather than thermodynamic immiscibility. The absence of residual crystallinity at 20% drug loading when processed at 150 °C demonstrates that the SOL-TEC matrix is thermodynamically capable of dissolving IND at this concentration. The presence of crystallinity at 110 °C therefore arises from insufficient molecular diffusion and incomplete dissolution within the available residence time. At lower temperatures, melt viscosity is elevated, reducing IND mobility and limiting interdiffusion into the polymer matrix. Under these conditions, undissolved crystalline domains may persist as kinetically trapped remnants. In contrast, higher temperature exponentially enhances diffusion coefficients and reduces viscosity, facilitating complete drug dissolution and molecular dispersion. This distinction between kinetic amorphization efficiency and thermodynamic miscibility is critical. It demonstrates that processing temperature defines a critical thermal input threshold required to achieve complete amorphization at a given drug loading. Exceeding this threshold ensures molecular-level homogeneity; falling below it results in partial crystalline retention despite overall compatibility of the system.

**Figure 6:**
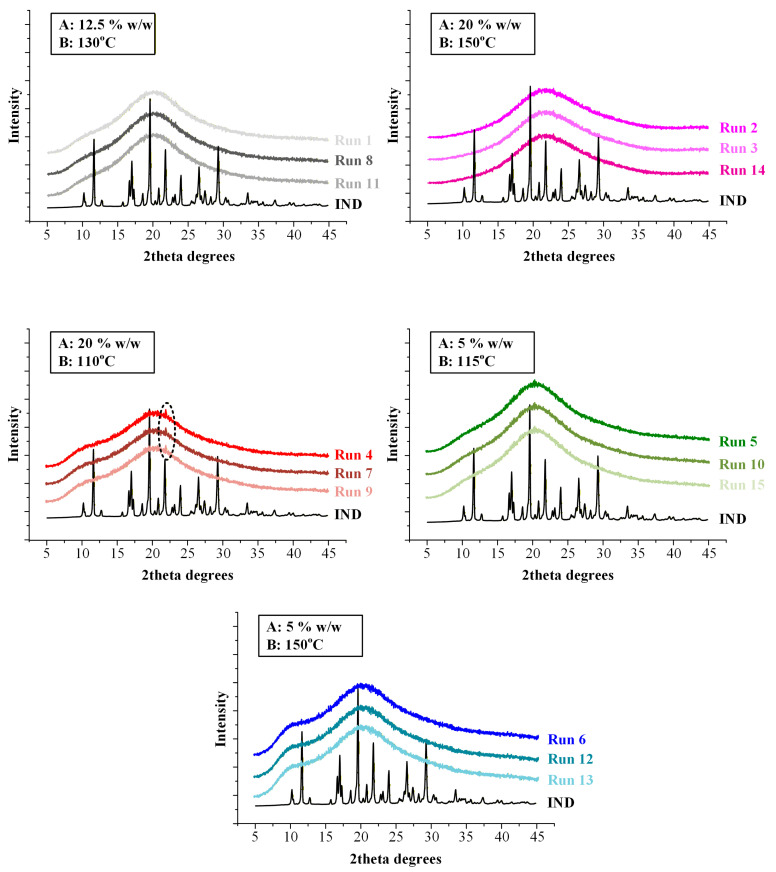
XRD diffractograms of pure IND and FILs corresponding to the individual experimental runs of the DoE (run numbers indicated in the figures). Diffractograms are grouped according to drug loading (A: 5%, 12.5%, and 20% *w*/*w* IND) and extrusion temperature (B: 110 °C, 130 °C, and 150 °C).

X-ray diffraction (XRD) analysis was performed on FILs obtained from all fifteen experimental runs in order to independently verify the solid-state form of IND following HME and to corroborate the DSC-based assessment of residual crystallinity. The obtained diffractograms are presented at Figure 6.

Crystalline IND (form γ) exhibited a series of sharp and well-defined reflections at 2θ values of 11.6°, 16.7°, 19.6°, 21.8°, 26.6°, and 29.3°, consistent with previously reported diffraction patterns [42]. These reflections served as reference markers for identification of crystalline domains within the extruded FILs.

FILs corresponding to Runs 4, 7, and 9 (20% *w*/*w* IND processed at 110 °C) exhibited a distinct diffraction peak at approximately 2θ ≈ 22°, which aligns with one of the characteristic reflections of crystalline IND. The presence of this peak confirms that a fraction of the drug retained long-range lattice order under these processing conditions. The absence of additional prominent reflections suggests that the crystalline fraction was limited and/or partially disordered, consistent with low residual crystallinity levels (~7.5%) quantified by DSC analysis.

In contrast, diffractograms of FILs obtained from the remaining twelve runs displayed only broad halo-type scattering patterns without detectable sharp reflections attributable to IND, indicating that the drug was present in an amorphous form within the SOL-TEC matrix.

Importantly, the use of XRD was critical to exclude the possibility of in situ amorphization during DSC heating. In partially crystalline systems, the melting endotherm observed in DSC can be underestimated or even suppressed due to rapid dissolution of residual crystallites within the polymer matrix during thermal scanning. The detection of a characteristic diffraction peak at ~22° in Runs 4, 7, and 9 confirms that crystalline domains were already present in the extruded FILs. Conversely, the absence of diffraction peaks in the remaining runs supports the conclusion that complete amorphization was achieved during extrusion when sufficient thermal input was applied.

Overall, DSC and XRD confirm that residual crystallinity occurs only at high drug loading and low temperature and results from kinetically limited drug dissolution during extrusion rather than thermodynamic incompatibility.

Generally, the DoE analysis revealed pronounced interdependencies between formulation composition, extrusion temperature, and sustainability-related process metrics. The investigated responses (Y_1_–Y_4_) were not independent but rather represented coupled manifestations of underlying thermo-rheological and molecular phenomena governing melt processing. In particular, extrusion temperature emerged as a dominant process variable simultaneously affecting electrical energy demand, drug amorphization efficiency, encapsulation performance, and material recovery.

Increasing thermal input enhanced molecular mobility within the polymeric matrix, reducing the risk of residual crystallinity. However, this benefit was accompanied by increased electrical energy consumption, reflecting the intrinsic trade-off between physicochemical performance and environmental burden. These findings underscore the necessity of multi-objective optimization strategies in melt-based pharmaceutical manufacturing, where thermal conditions must be carefully balanced to ensure complete amorphization while avoiding excessive energy expenditure.

Importantly, the explicit incorporation of electrical energy consumption (Y_1_) as a quantitative response within the DoE framework extends traditional formulation optimization beyond product-centric metrics. By treating energy demand as an intrinsic process attribute rather than an external consideration, the present approach enables rational identification of operating conditions that align product quality requirements with sustainability objectives.

#### 2.4.1. Statistical Diagnostics Tools for HME-DoE

To further assess the adequacy of the model, key statistical diagnostic metrics were evaluated. Table 3 presents the goodness-of-fit indicators, including R^2^, adjusted R^2^, predicted R^2^, and adequate precision for each response in the design. The predicted R^2^ values are in reasonable agreement with the corresponding adjusted R^2^ values, with differences less than 0.2, indicating a robust predictive capability of the model. Adequate precision, which reflects the signal-to-noise ratio, exceeds the recommended threshold of 4 for all responses, confirming that the model provides an adequate signal.

In addition, diagnostic plots are presented in the Supplementary Information (Figure S3) for response Y_1_ as a representative example, including the normal probability plot of residuals, residuals versus predicted values, residuals versus run order, and Cook’s distance plot. The normal probability plot shows that residuals closely follow the reference line, indicating approximate normality. The residual versus predicted plot displays no systematic pattern, supporting homoscedasticity and absence of model misspecification. The residuals versus run order plot shows no discernible trends, confirming independence of errors. Cook’s distance values remain below the critical threshold, indicating the absence of influential observations. Similar diagnostic behavior was observed for all other responses, confirming that the underlying regression assumptions are consistently satisfied across the design.

#### 2.4.2. Multi-Response Design Space and Sustainability-by-Design Selection

To translate the multivariate DoE outcomes into a practically applicable processing window, overlay contour analysis was conducted using predefined acceptance criteria reflecting both performance and sustainability targets: electrical energy consumption (Y_1_) ≤ 0.17 kWh, extrusion yield (Y_2_) ≥ 30%, encapsulation efficiency (Y_3_) ≥ 90%, and residual crystallinity (Y_4_) < 0.5%.

**Figure 7:**
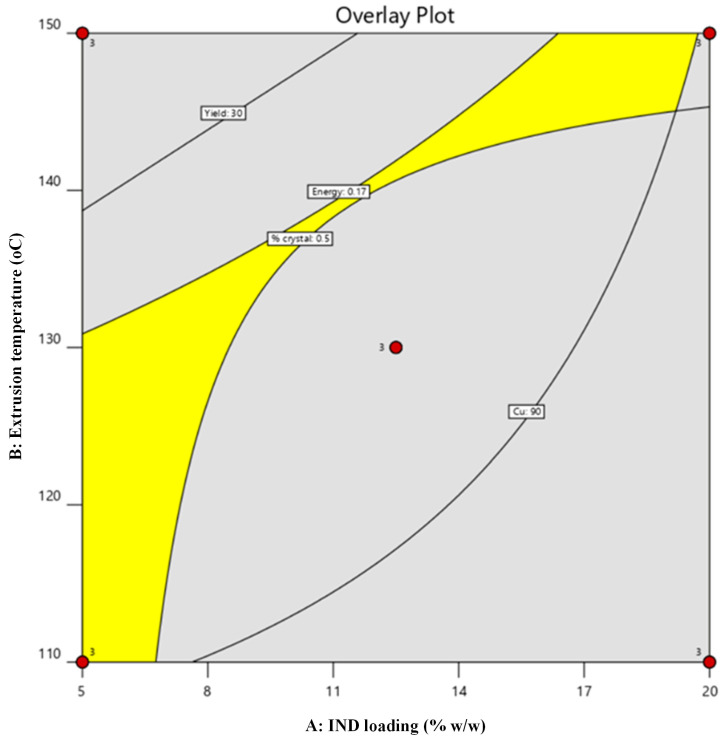
Multi-response overlay contour plot illustrating the optimized design space derived from the factorial DoE. The yellow region represents the operating window satisfying the predefined acceptance criteria.

The resulting composite design space (Figure 7) delineated a feasible region in which all responses simultaneously satisfied the specified constraints. Within this region, extrusion conditions provided sufficient thermal input to ensure molecular dispersion of IND and acceptable FIL manufacturability, while maintaining reduced electrical demand. The identified processing window therefore represents a sustainability-by-design operating envelope, in which energy efficiency is harmonized with material performance and downstream FDM suitability.

This integrated response-surface strategy demonstrates that environmental impact can be quantitatively embedded into pharmaceutical process optimization, enabling informed selection of extrusion parameters that minimize resource intensity without compromising solid-state quality attributes.

### 2.5. Selection of the Optimized FIL

The overlay analysis defines a constrained design space in which all responses meet the specified criteria, highlighting the interplay between drug loading, processing temperature, and performance attributes. As shown in Figure 7, the feasible region narrows considerably with increasing IND loading, particularly above ~10–15% *w*/*w*, where higher extrusion temperatures are required to maintain complete amorphization. This behavior reflects kinetic limitations in drug dissolution within the polymer matrix, as higher drug fractions increase the demand for molecular dispersion within the available residence time. Consequently, higher drug loadings are associated with a trade-off between amorphization efficiency and energy input, with regions of the design space excluded due to either residual crystallinity or increased energy consumption. In contrast, lower drug loadings provide a broader and more robust processing window.

Based on the multi-response overlay analysis, a formulation located within the identified feasible design space was selected as the optimized FIL composition for subsequent FDM 3D printing studies. The selected operating point corresponded to a composition containing 5% *w*/*w* IND, 10% *w*/*w* TEC, and 85% *w*/*w* SOL, processed at an extrusion temperature of 130 °C.

This specific formulation–temperature combination was chosen because it simultaneously satisfied all predefined performance and sustainability criteria, including complete drug amorphization, high encapsulation efficiency, acceptable extrusion yield, and reduced electrical energy consumption. Importantly, this condition provided sufficient thermal input to ensure molecular dispersion of IND while avoiding excessive energy demand associated with higher extrusion temperatures. This optimum point should be interpreted within the boundaries of the investigated design space rather than as a fundamental limitation of the formulation system.

The selection of this optimized FIL was not intended as an endpoint of process development but rather as a rationally validated intermediate platform for downstream additive manufacturing. Given that FDM printing represents a secondary thermal processing step, it was essential to first establish a FIL composition that ensures solid-state stability, compositional uniformity, and controlled thermo-mechanical behavior during extrusion. The validated FIL therefore served as the foundational material for subsequent optimization of FDM. By establishing an HME processing window that balances molecular dispersion efficiency with energy consumption, the present approach ensures that downstream FDM optimization can be performed on a formulation that is both physicochemically robust and environmentally rational.

Following its selection from the multi-response design space, the optimized FIL formulation (5% *w*/*w* IND, 10% *w*/*w* TEC, processed at 130 °C) was experimentally prepared and systematically evaluated to validate the predictive accuracy of the DoE model. All four critical responses—electrical energy consumption (Y_1_), extrusion yield (Y_2_), encapsulation efficiency (Y_3_), and residual crystallinity (Y_4_)—were determined using the methodologies described previously.

The experimentally measured electrical energy consumption (Y_1_) was 0.1625 kWh, as calculated based on the recorded voltage and current intensity during extrusion of the optimized FIL (Figure S3), closely matching the model-predicted value (~0.169 kWh). This agreement confirms the reliability of incorporating electrical demand as a quantitative response within the optimization framework.

The experimentally determined extrusion yield (Y_2_) was 33%, in excellent agreement with the predicted value (~32%), indicating that the selected processing temperature provided an appropriate balance between melt flow and material recovery.

Encapsulation efficiency (Y_3_) was 98.03%, as determined from three independent FIL segments, demonstrating homogeneous drug distribution and efficient molecular dispersion within the polymeric matrix. The slight deviation from the predicted 100% value falls within normal analytical variability and does not indicate formulation inconsistency.

**Figure 8:**
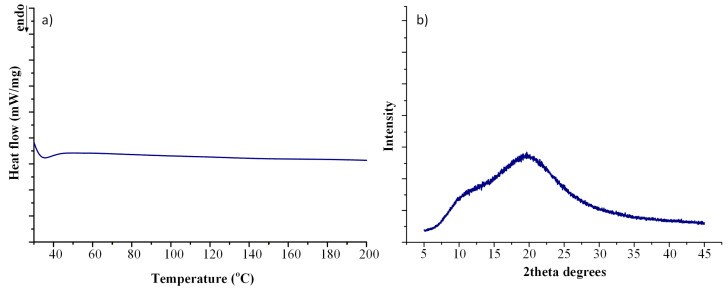
(**a**) DSC thermogram and (**b**) XRD diffractogram of the optimized FIL.

Solid-state characterization confirmed complete drug amorphization. The obtained DSC thermogram (Figure 8a) showed no detectable melting endotherm corresponding to crystalline IND, while the XRD diffractogram (Figure 8b) exhibited a diffuse halo pattern without characteristic crystalline reflections. Residual crystallinity (Y_4_) was therefore calculated as 0% within the detection limits of the applied techniques.

Collectively, the close agreement between predicted and experimentally determined values for Y_1_–Y_4_ validates the robustness of the multi-response DoE model and confirms that the selected operating point simultaneously satisfies performance and sustainability criteria. This experimental confirmation strengthens the proposed sustainability-by-design strategy for melt-processed ASD development.

**Figure 9:**
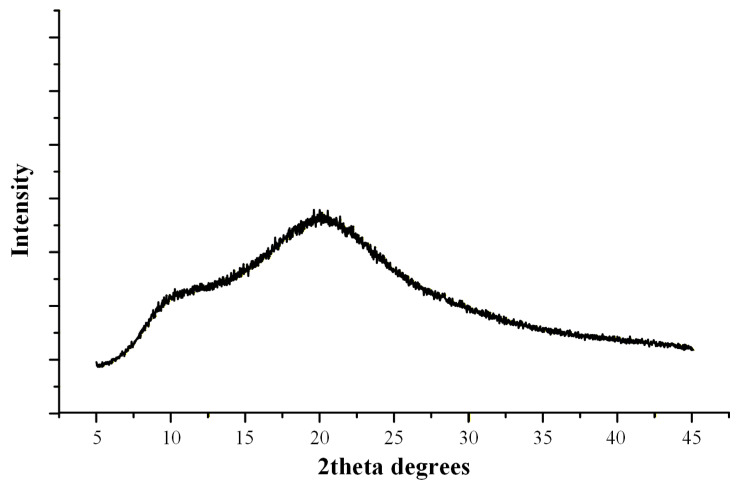
XRD diffractogram of the optimized FIL after storage at 25 °C/60% RH for 6 months.

To assess the physical stability of the optimized ASD system, the FIL was stored at 25 °C/60% RH for 6 months (6 M) and subsequently analyzed by XRD (Figure 9). The diffractograms showed no evidence of recrystallization, as no characteristic diffraction peaks of crystalline IND were detected after storage. This indicates that the amorphous state of the drug was preserved over the studied period. The observed stability suggests that the system remains kinetically stabilized within the polymer matrix.

### 2.6. FDM Process Optimization

#### 2.6.1. Selection of the Optimal Dosage Form Geometry

The influence of dosage form geometry on FDM process performance was systematically evaluated using the optimized IND-loaded FIL. Three geometries—capsule, cylindrical tablet, and torus—were printed under identical processing conditions to isolate the structural contribution to electrical energy consumption, printing duration, and mass accuracy. The 3D-printed dosage forms with the different geometries are shown in Figure S5. Table 4 summarizes the printing energy, printing time, and weight yield for each printed dosage form. Electrical energy consumption was calculated from the recorded voltage (Figure S6) and current intensity (Figure S6) throughout the printing process for each geometry. Total electrical energy consumption was determined using the equation W=V×I, where *V* represents the applied voltage and *I* the current intensity over the entire printing duration. Printing time was directly provided by the Flashforge slicing software (FlashPrint 5, version 5.6.0) for each geometry. Weight yield was defined as the ratio between the experimentally measured mass of the printed dosage form and its theoretical mass, serving as an indicator of deposition accuracy and dimensional fidelity.

Although identical FIL mass was used for all geometries, measurable differences in total electrical energy consumption were observed. The torus geometry exhibited the highest energy demand (0.1608 kWh), followed by the capsule (0.1498 kWh), while the cylindrical tablet required the lowest energy input (0.1498 kWh). To further quantify the influence of geometry on process efficiency, energy consumption was normalized to the mass of printed material (kWh/g). The calculated values were 0.297 kWh/g for the capsule, 0.283 kWh/g for the cylindrical tablet, and 0.344 kWh/g for the torus geometry. The cylindrical geometry exhibited the lowest energy consumption per unit mass, indicating more efficient material deposition, while the torus geometry showed increased energy demand relative to the printed mass.

Because instantaneous electrical power during FDM printing is directly related to the mechanical load of FIL feeding, nozzle heating, and printhead motion, total energy consumption is influenced not only by material mass but also by printing time and motion dynamics. The torus geometry required the longest printing time (21 min 30 s), whereas the cylindrical tablet was completed in less time (18 min 25 s).

The increased energy demand of the torus can be mechanistically attributed to its structural complexity. Curvature transitions and the presence of an internal void require frequent changes in printhead direction and acceleration, increasing travel movements and localized deposition events. These dynamic motion adjustments prolong printing duration and elevate cumulative mechanical and thermal load. In contrast, the cylindrical tablet geometry presents continuous, symmetric deposition paths with minimal directional changes, enabling more efficient material deposition and reduced travel movements. From a sustainability perspective, the direct relationship between printing time and electrical demand indicates that geometric simplification can reduce the carbon footprint of additive manufacturing without altering formulation composition.

Weight yield—defined as the ratio between experimentally obtained mass and theoretical mass—serves as an indirect measure of deposition precision and dose accuracy. The cylindrical tablet exhibited the highest weight yield (0.88), followed by the capsule (0.84), whereas the torus demonstrated the lowest value (0.78). Reduced weight yield in the torus geometry likely arises from cumulative deposition inconsistencies in regions of curvature and internal bridging. Complex geometries may introduce minor over- or under-extrusion events due to transient pressure fluctuations within the melt channel during acceleration changes. Additionally, internal void formation may reduce effective material packing density at constant infill settings. The superior mass accuracy observed in the cylindrical tablet reflects uniform layer stacking, consistent perimeter overlap, and minimal structural stress during cooling. From a pharmaceutical standpoint, improved weight yield directly translates to enhanced dose reproducibility and reduced variability.

Importantly, this evaluation demonstrates that geometry selection in FDM-based pharmaceutical manufacturing should not be driven solely by aesthetic preferences or conventional tablet shapes, but rather by integrated sustainability and process-performance criteria. When energy consumption, printing time, mass accuracy, and normalized energy demand are evaluated collectively, the cylindrical tablet geometry emerges as the most efficient and robust configuration. In addition to exhibiting shorter printing duration and higher mass accuracy, the cylindrical tablet demonstrated the lowest energy consumption per unit mass (0.283 kWh/g), compared to the capsule (0.297 kWh/g) and torus (0.344 kWh/g) geometries. These results indicate superior deposition efficiency and reduced non-productive movements, consistent with the simpler and more continuous too path associated with the cylindrical design. On this basis, the cylindrical tablet was selected as the optimal dosage form for subsequent FDM process optimization studies, ensuring that further parameter refinement would be conducted using a geometrically robust and energy-efficient platform.

#### 2.6.2. DoE-Driven Optimization of FDM 3D-Printing Process

Following selection of the cylindrical tablet as the most efficient geometry, a full factorial DoE was applied to quantify the influence of key FDM processing parameters on printing time (R_1_), electrical energy consumption (R_2_), and IND encapsulation efficacy (R_3_). Platform temperature (X_1_), nozzle temperature (X_2_), and printing speed (X_3_) were examined within practical operating ranges, and the corresponding experimental matrix and response values are presented in Table 5, with ANOVA outcomes summarized in Table 6. Regression modeling produced coded-factor equations describing the response surfaces within the investigated design space.

Printing time (R_1_) ranged from 0.194 to 0.246 h. ANOVA identified platform temperature and nozzle temperature as statistically significant main effects (*p* = 0.0003 and *p* < 0.0001, respectively), while the X_1_X_2_ interaction was also significant (*p* = 0.0012), indicating that the temperature dependence of printing time is not purely additive. Consistent with these findings, the coded regression equation for R_1_ (R_1_ = 0.2250 + 0.0102*X_1_ − 0.0030*X_2_ − 0.0001*X_1_X_2_) shows a positive coefficient for platform temperature and negative coefficients for nozzle temperature. Mechanistically, increasing nozzle temperature reduces melt viscosity in the liquefier zone and stabilizes extrusion flow, thereby reducing time penalties associated with intermittent under-extrusion or flow interruptions. In contrast, higher platform temperature can prolong thermal equilibration and layer stabilization, which may manifest as slightly longer effective printing times depending on the thermal history of deposition.

Electrical energy consumption (R_2_) varied between 0.026 and 0.048 kWh per printing run and was strongly sensitive to all three factors (Table 6). It should be noted that within the DoE framework, electrical energy consumption was defined as the total energy required per printing process (kWh per print), encompassing all contributions, including bed heating, nozzle heating, and steady-state operation during printing. Unlike the preliminary geometry study, where all samples were produced under identical conditions and normalization (kWh/g) enabled direct comparison of structural effects, the DoE involves systematic variation in process parameters. This approach was intentionally adopted to capture the overall process-level energy demand, ensuring a direct linkage between processing parameters (e.g., temperature settings and printing speed) and energy consumption. The voltage and current intensity profiles recorded during the 3D printing process are presented in Figures S8 and S9, respectively. These profiles provide real-time insight into the electrical load of the system under each DoE condition, reflecting the combined thermal and mechanical demands imposed by the selected platform temperature, nozzle temperature, and printing speed. Platform temperature exhibited the dominant contribution (F = 2560.97, *p* < 0.0001), reflecting the continuous baseline power required to maintain the build plate at elevated setpoints. Nozzle temperature and printing speed were also significant contributors (*p* < 0.0001). The coded equation for R_2_ (R_2_ = 0.0376 + 0.0084*X_1_ + 0.0011*X_2_ − 0.0010*X_3_) indicates that increases in platform and nozzle temperatures increase energy demand, whereas increased printing speed decreases cumulative energy consumption. This reflects the time-integrated nature of energy: although higher speeds may not reduce instantaneous power substantially, they shorten the total printing duration, thereby reducing the total energy consumption over time.

IND encapsulation efficacy (R_3_) ranged from 85.2% to 96.4%. Both platform and nozzle temperatures were highly significant (*p* < 0.0001), and multiple interaction terms (X_1_X_2_, X_1_X_3_) were significant, demonstrating a non-linear coupling between thermal boundary conditions and dose accuracy. The coded equation for R_3_, R_3_ = 92.53 + 0.9919*X_1_ + 1.54*X_2_ − 2.11*X_1_X_2_ + 1.51*X_1_X_3_), suggests that, in isolation, higher platform and nozzle temperatures increase encapsulation efficacy values, consistent with improved interlayer fusion and reduced voiding that can otherwise lead to mass deficits and under-dosing. However, the negative Χ_1_Χ_2_ term indicates that simultaneously elevating both temperatures can offset these gains. Within the investigated range, printing speed did not exhibit a significant main effect on assay (Table 6), implying that deposition remained sufficiently stable to maintain dose accuracy, provided thermal conditions were appropriately selected.

Collectively, the coded equations and ANOVA demonstrate that FDM performance is governed by coupled thermo-mechanical mechanisms. Platform temperature primarily dictates baseline electrical demand and interlayer thermal history; nozzle temperature modulates melt rheology and extrusion stability; and printing speed influences the time-dependent integration of electrical power and overall throughput. These findings support the necessity of multi-objective optimization to identify an operating window that minimizes energy and printing time without compromising assay performance.

#### 2.6.3. Statistical Diagnostics Tools for FDM 3D Printing

To further evaluate the suitability of the model, several statistical diagnostic measures were examined once again. As summarized in Table 7, the goodness-of-fit parameters—namely, R^2^, adjusted R^2^, predicted R^2^, and adequate precision—were calculated for each response variable. The predicted R^2^ values show good consistency with the adjusted R^2^ values, with deviations remaining below 0.2, which demonstrates a satisfactory predictive performance. In addition, the adequate precision values for all responses are greater than 4, indicating an acceptable signal-to-noise ratio and confirming that the model provides a reliable signal.

Diagnostic plots are provided in the Supplementary Information (Figure S10) for response R_1_ as a representative example, including the normal probability plot of residuals, residuals versus predicted values, residuals versus run order, and Cook’s distance plot. The normal probability plot shows that residuals closely follow the reference line, supporting approximate normality. The residuals versus predicted values plot displays no systematic pattern, indicating homoscedasticity and absence of model misspecification. The residuals versus run order plot shows no discernible trends, confirming independence of errors. Furthermore, all Cook’s distance values remain below the critical threshold, indicating the absence of influential observations. Similar diagnostic behavior was observed for all other responses, confirming that the regression assumptions are consistently satisfied across the design space.

#### 2.6.4. Multi-Response Design Space and Sustainability-by-Design Selection for FDM 3D Printing

To translate the multivariate DoE outcomes into a practically applicable FDM processing window, overlay contour analysis was conducted using predefined acceptance criteria reflecting both manufacturing efficiency and sustainability targets: printing time (R_1_) < 0.22 h, electrical energy consumption (R_2_) < 0.035 kWh per print, and IND loading efficacy (R_3_) > 91%.

The composite overlay generated from the response surface models delineated a constrained yet clearly defined feasible region in which all three criteria were simultaneously satisfied. Within this region, the selected combinations of platform temperature, nozzle temperature, and printing speed provided sufficient thermal input to ensure accurate material deposition and dose uniformity, while maintaining reduced electrical demand and shortened production time.

**Figure 10:**
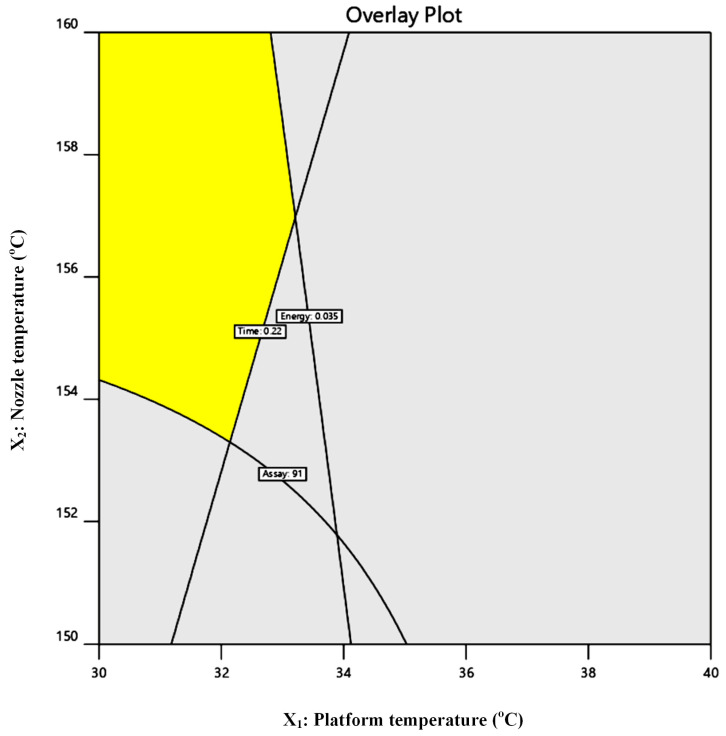
Multi-response overlay contour plot illustrating the optimized FDM design space derived from the factorial DoE. The yellow region represents the operating window satisfying the predefined acceptance criteria: printing time (R_1_) < 0.22 h, electrical energy consumption (R_2_) < 0.035 kWh per print, and IND loading efficacy (R_3_) > 91%.

Mechanistically, the feasible design space corresponded to moderate platform temperatures, which limited baseline heater load, combined with sufficiently elevated nozzle temperatures to promote stable melt flow and interlayer fusion. Simultaneously, intermediate-to-high printing speeds reduced cumulative process duration without compromising deposition fidelity. Outside this region, either excessive thermal input increased electrical demand beyond the predefined sustainability threshold, or insufficient thermal energy compromised assay values, likely due to suboptimal melt homogenization and interlayer bonding (Figure 10).

To experimentally verify the predictive capability of the FDM DoE model, one operating point located within the optimized multi-response design space was selected for confirmation. This point corresponded to a platform temperature of 32 °C, a nozzle temperature of 158 °C, and a printing speed of 11 mm/s. These conditions were identified from the overlay contour analysis as satisfying simultaneously the predefined criteria of minimized printing time, reduced electrical energy consumption, and acceptable IND assay.

According to the regression models derived from the factorial design, the predicted responses at this operating point were 0.21 h for printing time (R_1_), 0.032 kWh for electrical energy consumption (R_2_), and 93.3% for drug loading efficacy (R_3_). To validate these predictions, three cylindrical tablets were printed under the proposed optimal conditions. All responses were calculated as the mean of the three independent prints.

The experimentally recorded printing time, obtained directly from the FDM printer software (FlashPrint 5, version 5.6.0), was 0.202 h. This value closely matches the model-predicted time of 0.21 h, confirming the reliability of the regression model for R_1_ within the defined design space.

Voltage and current intensity profiles recorded during printing under the optimized conditions are presented in the Supplementary Material (Figure S11). The experimentally determined energy consumption was consistent with the predicted value (~0.032 kWh), indicating that the selected parameter combination effectively limits cumulative electrical demand. The relatively low platform temperature reduces baseline heater load, while the elevated nozzle temperature ensures stable melt flow and continuous deposition. The selected printing speed further contributes to energy reduction by shortening total process duration without compromising deposition fidelity.

The experimentally determined IND assay was 92%, in close agreement with the predicted value of 93.3%. This confirms that the selected thermal and kinematic conditions provide sufficient melt homogenization and interlayer fusion to maintain dose accuracy. The minor deviation between predicted and observed values falls within expected analytical variability and does not indicate model inadequacy.

Overall, the close agreement between predicted and experimental values for R_1_, R_2_, and R_3_ validates the robustness of the FDM DoE model and confirms that the identified processing window effectively balances manufacturing efficiency, energy minimization, and pharmaceutical performance. This final validation step demonstrates that sustainability-driven parameter selection can be quantitatively integrated into additive pharmaceutical manufacturing without compromising dosage quality.

#### 2.6.5. Dissolution Studies of Optimized Dosage Forms

**Figure 11:**
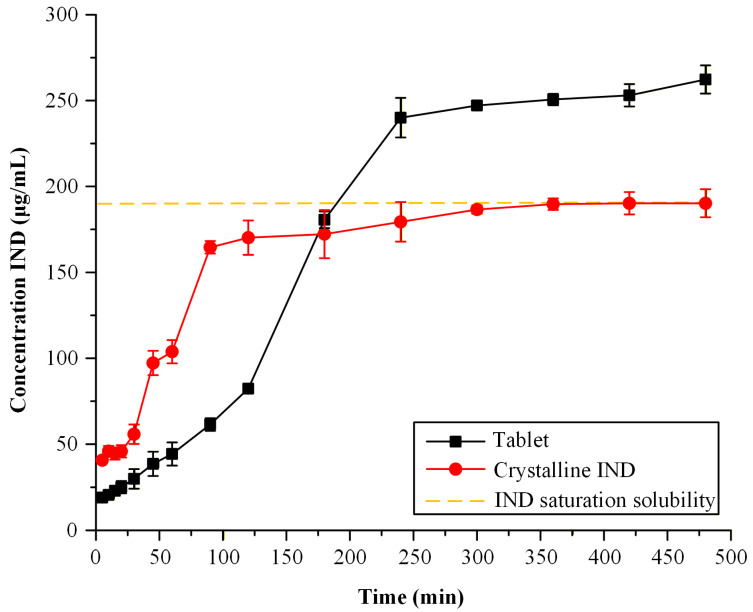
Dissolution profiles of crystalline IND and optimized ASD-based 3D-printed tablets in PBS (pH 6.8) at 37 °C. The orange dashed line represents the saturation solubility of crystalline IND in the dissolution medium. Data are presented as mean ± standard deviation (*n* = 3).

To evaluate the effectiveness of the optimized 3D-printed tablets in enhancing drug solubility, dissolution studies were performed in phosphate-buffered solution (PBS, pH 6.8) and compared with crystalline IND. The dissolution profiles (Figure 11) demonstrate a significant enhancement in drug release from the ASD-based printed dosage forms compared to crystalline IND. The crystalline drug exhibited limited dissolution, consistent with its low aqueous solubility and dissolution rate-limited behavior, reaching a plateau at approximately 190 μg/mL after 300 min, corresponding to its saturation solubility in the selected medium [43].

In contrast, the ASD formulations exhibited a slower initial drug release during the first 180 min, with lower dissolved concentrations compared to crystalline IND. However, at later time points, the ASD systems achieved higher dissolved concentrations, exceeding the apparent solubility of the crystalline drug. This behavior is consistent with the amorphous state of IND within the polymer matrix, which eliminates the energetic barrier associated with crystal lattice disruption. The delayed release phase may be attributed to matrix-controlled drug diffusion and polymer hydration kinetics, which govern the initial release behavior.

At extended time scales, the increased molecular mobility and higher apparent solubility of the amorphous form enable the generation of supersaturated solutions, while the presence of the polymer likely inhibits recrystallization and supports the maintenance of supersaturation. These results confirm that the optimized HME-FDM process produces ASD systems capable of enhancing apparent solubility, despite differences in early-stage release kinetics.

## 3. Discussion

The present study establishes a formulation-process integrated framework in which pharmaceutical performance and process-level energy demand are simultaneously optimized in melt-based additive manufacturing of ASDs. Unlike prior studies that treat energy consumption as a secondary engineering outcome, the current approach embeds electrical energy demand as a quantitative response within a dual-stage DoE strategy, thereby enabling direct coupling between molecular-level phenomena and process efficiency.

The selection of the SOL-TEC system was mechanistically justified through melt miscibility behavior and solid-state analysis. The formation of a homogeneous molten phase in the IND-SOL-TEC system indicates favorable intermolecular interactions and sufficient thermodynamic compatibility to support molecular dispersion of the drug. Residual crystallinity was confined to the high drug loading-low temperature region of the design space, demonstrating that incomplete amorphization arose from kinetic limitations rather than intrinsic immiscibility. Specifically, incomplete amorphization arises when molecular mobility is insufficient to support drug diffusion into the polymer matrix within the available residence time. Increasing thermal input enhances segmental mobility and facilitates dissolution of drug domains, consistent with diffusion-controlled amorphization mechanisms in melt systems.

This distinction between thermodynamic miscibility and kinetic amorphization efficiency is critical for rational ASD development via melt technologies and underscores the importance of carefully defining the thermal processing window.

A key conceptual advancement of this work lies in treating electrical energy demand as an intrinsic process attribute rather than an external environmental descriptor. It should be emphasized that, within the scope of the present study, electrical energy consumption is interpreted as a process-level indicator of sustainability rather than a comprehensive measure of environmental impact. While electrical energy data can, in principle, be translated into carbon emissions using established conversion factors, such as CO_2_ equivalents per unit of energy, the magnitude of this conversion is inherently dependent on the electricity generation mix and geographical context. Accordingly, the present framework should be interpreted as an energy-informed optimization approach, providing a foundation for future integration with carbon footprint analysis and life-cycle assessment methodologies. During HME, energy consumption originates from both thermal heating and mechanical work associated with melt transport. The observed reduction in electrical demand with increasing drug loading can be interpreted as a consequence of drug-induced viscosity modulation, whereby IND behaves as a low-molecular-weight component that perturbs polymer–polymer interactions and reduces entanglement density, thereby lowering shear resistance and mechanical torque. This interpretation is further supported by literature reports indicating that drug incorporation into SOL can induce Τg depression and increase molecular mobility, which are associated with plasticization effects and reduced resistance to molecular rearrangement during processing. However, it should be emphasized that viscosity-related interpretations in the present study are based on indirect evidence derived from process behavior and energy consumption trends and were not directly confirmed through rheological measurements. In contrast, increasing extrusion temperature elevates heater load and thermal losses, resulting in greater electrical demand. The response surface analysis thus reveals a measurable trade-off between thermal input required for amorphization and the environmental cost of that input.

A similar coupling between thermal parameters and electrical demand was observed during FDM processing. Platform temperature emerged as the dominant contributor to energy consumption, reflecting the continuous baseline heating requirement of the build plate. Nozzle temperature modulated melt rheology and deposition stability, while printing speed primarily influenced energy through time integration effects. Because electrical energy represents the integral of instantaneous power over time, reductions in printing duration directly translate into lower cumulative energy demand, even when instantaneous power remains comparable. This reinforces the importance of interpreting energy consumption as a time-dependent quantity rather than as a static power measurement.

The geometry screening phase further demonstrated that dosage form architecture influences sustainability metrics independently of formulation composition. The cylindrical tablet geometry minimized printing time and electrical demand while maximizing mass accuracy, owing to its continuous deposition paths and reduced need for directional changes. More complex geometries introduced additional travel movements and acceleration events, increasing cumulative mechanical load and energy consumption. This indicates that process efficiency is not solely a function of material behavior but is also governed by motion kinematics and path planning, linking digital design directly to energy demand.

The dual-stage multi-response overlay analyses for both HME and FDM identified operating envelopes in which complete amorphization, acceptable dose accuracy, and reduced electrical demand were simultaneously achieved. Experimental validation confirmed close agreement between predicted and observed responses, supporting the robustness of the statistical models and the feasibility of embedding sustainability metrics within pharmaceutical process optimization.

Within the broader context of advanced drug delivery manufacturing, this work contributes to the evolving paradigm in which digital fabrication technologies are evaluated not solely for their personalization capabilities but also for their environmental performance. By quantitatively integrating electrical energy consumption into the DoE framework, the study provides a reproducible methodology for aligning formulation science, process engineering, and sustainability objectives. Future investigations may expand this approach through integration of life-cycle assessment methodologies, long-term physical stability studies under optimized thermal histories, and evaluation of renewable energy sourcing scenarios. Generally, these efforts will be essential for establishing additive pharmaceutical manufacturing as both a performance-driven and environmentally responsible technology platform.

## 4. Materials and Methods

### 4.1. Materials

IND (form γ, purity ≥ 98%, CAS: 53-86-1) was obtained from Acros Organics (Geel, Belgium). Partially hydrolyzed PVA (Parteck^®^ MXP, CAS: 9002-89-5, lot no. F2016164812; hydrolysis degree 87–89%; average molecular weight approximately 32,000 Da) was obtained from Merck Millipore (Merck Millipore, Burlington, MA, USA). SOL, a polyvinyl caprolactam-polyvinyl acetate-polyethylene glycol graft copolymer (CAS: 402932-23-4), was kindly supplied by BASF (Ludwigshafen, Germany) and evaluated as an alternative polymeric carrier for ASD development. MAN (Pearlitol^®^ 160C, D-mannitol, CAS: 69-65-8, lot no. E833T) was purchased from Roquette (Lestrem, France). SUC (Compressuc^®^ PS, CAS: 57-50-1, lot no. 0175123241) was obtained from BeghinSay, Tereos Internacional (Lille, France). TEC (Citrofol^®^ AI, CAS: 77-93-0, lot no. 3038933) was supplied by Jungbunzlauer Ladenburg GmbH (Ladenburg, Germany). CA (CAS: 77-92-9, lot no. K48576902) was obtained from Merck & Co. (Rahway, NJ, USA). PEG6000 (CAS: 25322-68-3) was kindly donated by Rontis S.A. (Athens, Greece). All materials were used as received without further purification.

### 4.2. TGA Studies

TGA was conducted to assess the thermal stability of the raw materials prior to melt-based processing and to support the selection of suitable formulation components for HME and FDM. Measurements were performed using a Shimadzu TGA-50 thermogravimetric analyzer (Shimadzu, Tokyo, Japan). Approximately 5 mg of each sample (IND, PVA, SOL, TEC, PEG 6000, MAN, and CA) was accurately weighed and placed in aluminum sample pans attached to the microbalance assembly. Samples were heated from 25 °C to 300 °C at a heating rate of 10 °C/min under a nitrogen purge atmosphere (flow rate: 50 mL/min). Mass change was recorded as a function of temperature, and thermograms were analyzed to identify the onset and extent of mass loss events relevant to thermal processing suitability.

### 4.3. Miscibility Studies via HSM

#### 4.3.1. Miscibility Screening of Polymeric Carriers and Plasticizers

The miscibility of the investigated polymeric carriers, namely, PVA and SOL, with selected plasticizers (MAN, PEG6000, CA, and TEC), was initially evaluated using HSM. PMs of each polymer–plasticizer pair were prepared at a polymer-to-plasticizer ratio of 70:30 *w*/*w*. This high plasticizer content was selected to enable reliable visual assessment of phase behavior and detection of potential demixing or residual crystalline domains under thermal conditions. The mixtures were heated from 25 °C to complete melting at a heating rate of 10 °C/min.

Observations were conducted using a polarized optical microscope (BX41, Olympus, Tokyo, Japan) equipped with a Linkam THMS600 heating stage (Linkam Scientific Instruments Ltd., Surrey, UK). Miscibility assessment was performed via direct visual observation of the molten systems under polarized light, focusing on the formation of homogeneous melts and the presence or absence of phase separation.

#### 4.3.2. Miscibility Screening of Drug–Polymer–Plasticizer Systems

Polymer–plasticizer systems that demonstrated miscibility were subsequently evaluated for their compatibility with IND. PMs of IND with the selected polymer–plasticizer combinations were prepared at a drug:polymer:plasticizer ratio of 60:20:20 *w*/*w*. This composition was intentionally selected to impose a high drug loading, enabling the assessment of melt-state compatibility and the ability of the system to accommodate the drug without phase separation or residual crystallinity. The mixtures were heated from 25 °C to complete melting at a heating rate of 10 °C/min using the same HSM setup described above. Miscibility was assessed through direct visual observation of the molten systems under polarized light.

### 4.4. Preparation of FILs and Preliminary Plasticizer Optimization

Preliminary HME studies were conducted to evaluate the influence of plasticizer concentration on FIL formation and handling characteristics. PMs consisting of IND, the selected polymeric carrier, and the plasticizer were prepared with a fixed drug loading of 10% *w*/*w*, while the plasticizer concentration was varied at 5, 10, and 15% *w*/*w*. The corresponding polymer content was adjusted accordingly to maintain the desired composition. Each formulation was prepared at a total batch mass of 30 g and manually blended to ensure homogeneity prior to extrusion.

Hot-melt extrusion was performed using a single-screw extruder (Noztek Touch, Noztek Ltd., Shoreham-by-Sea, UK). Processing temperatures were set within the range of 110–160 °C, selected to ensure sufficient softening of the polymeric matrix and melting of the active pharmaceutical ingredient while remaining within a thermally safe processing window. The screw rotation speed was fixed at 50 rpm. Extrusion was carried out using a stainless-steel nozzle with a diameter of 1.75 mm to produce drug-loaded FILs intended for subsequent FDM processing.

The extruded FILs were collected and visually inspected to assess continuity of extrusion, uniformity of appearance, and handling behavior during collection and manipulation. Observations from these preliminary trials were used to guide the selection of a suitable plasticizer concentration for further formulation and process optimization studies.

### 4.5. DoE for HME Optimization

A systematic DoE approach was employed to quantify the influence of formulation and processing variables on FIL quality attributes and process sustainability metrics during HME. A full factorial 2^2^ design with a central point was implemented with two independent variables A: IND content (% *w*/*w*) and B: extrusion temperature (°C). Each factor was studied at three levels (low, medium and high), resulting in five unique experimental conditions. Specifically, IND content was set at 5, 12.5 and 20% *w*/*w*, and extrusion temperature varied at 110, 130, and 150 °C. The selected factor ranges were defined to (i) remain within the thermally safe processing window identified by TGA for the raw materials and (ii) to cover practically relevant HME conditions for the production of pharmaceutical FILs intended for downstream FDM. Experimental design generation and analysis were performed using Design-Expert software (version 6.0.4, Stat-Ease Inc., Minneapolis, MN, USA), with each experimental condition conducted in triplicate (15 total experimental runs) under fully randomized order to minimize systematic bias.

Four responses were evaluated: Y_1_, electrical energy consumed during extrusion; Y_2_, extrusion yield (%); Y_3_, IND encapsulation efficiency (%); and Y_4_, residual crystallinity (%). DoE modeling and analysis of variance (ANOVA) were performed at a 95% confidence level. Model adequacy was evaluated using standard diagnostic criteria (e.g., residual normality, residuals vs. predicted, leverage/Cook’s distance, lack-of-fit testing) and fit statistics (R^2^, adjusted R^2^, predicted R^2^, and adequate precision). Response surface methodology was used to visualize main effects and interactions, while overlay contour plots were employed to identify a design space satisfying multiple performance and sustainability criteria simultaneously.

#### 4.5.1. Electrical Energy Consumption During HME (Y_1_)

Electrical energy consumption during extrusion was recorded using an Electrocorder AL-2VA energy logger (Acksen Ltd., Newtownabbey, UK), suitable for monitoring electrical loads in domestic and light commercial applications. The device continuously measures voltage and current, and data were acquired at a sampling interval of 5 s. Energy consumption (Y_1_) was expressed as total electrical energy demand per extrusion run (KWh).

#### 4.5.2. Extrusion Yield (Y_2_)

Extrusion yield (Y_2_) was determined in order to evaluate the material recovery efficiency of the HME process. Specifically, extrusion yield was calculated as the ratio between the total mass of FIL successfully collected after extrusion and the initial mass of the PM introduced into the extruder hopper. The yield was expressed as a percentage according to the following equation:

(1) $$Extrusion\;yield\left(\%\right)=\frac{total\;mass\;of\;collected\;FIL}{initial\;mass\;of\;PM}*100$$

#### 4.5.3. Encapsulation Efficiency of IND in FILs (Y_3_)

Encapsulation efficiency (Y_3_) was determined by assaying IND content in FIL segments collected from three distinct locations along each FIL. Samples were dissolved completely in dimethylformamide (DMF). IND concentration was quantified by UV-Vis spectroscopy at 318 nm (Shimadzu, Kyoto, Japan). Quantification was performed using a calibration curve of IND in DMF over the range of 25–150 ppm, which exhibited excellent linearity (y = 0.0166x + 0.0851, R^2^ = 0.9999). The accuracy of the method was evaluated through recovery testing at representative concentration levels within the calibration range, yielding acceptable recovery values (98–100%). In addition, the photometric accuracy and linear dynamic range of the instrument were verified using potassium dichromate (K_2_Cr_2_O_7_) standard solutions, demonstrating reliable absorbance measurements up to 5 Abs. Method sensitivity was assessed by determining the limits of detection (LOD) and quantification (LOQ), calculated according to standard equations (LOD = 3.3σ/S and LOQ = 10σ/S, where σ is the standard deviation of the response and S is the slope of the calibration curve). The LOD and LOQ were estimated to be 0.6 ppm and 1.9 ppm, respectively, indicating high sensitivity of the analytical method. Encapsulation efficiency was calculated as:

(2) $$Encapsulation\;efficiency\left(\%\right)=\frac{experimental\;IND\;concentration}{theoretical\;IND\;concentration}*100$$

#### 4.5.4. Residual Crystallinity (Y_4_)

Residual crystallinity (Y_4_) was initially estimated by differential scanning calorimetry (DSC) using a DSC 204 F1 Phoenix (NETZSCH, Selb, Germany). Accurately weighed samples (~5.0 ± 0.1 mg) were sealed in aluminum pans and heated from 25 °C to 180 °C at 10 °C/min under nitrogen (50 mL/min). The melting point (T_m_) of the examined systems were determined as the highest (peak) temperature point and the temperature inflection point of the heat flow curve, while the enthalpy of fusion (ΔH_f_) was quantified as the total area under the heat flow curve. The standard deviations for both temperatures and enthalpies determined in this study did not exceed 1.0 °C and 3.0 J/g, respectively. Residual crystallinity was estimated by comparing the melting enthalpy of the FIL sample (ΔH_FIL_) to that of the corresponding PM (ΔH_PM_):

(3) $$Crystallinity\left(\%\right)=\frac{{\Delta H}_{FIL}}{{\Delta H}_{PM}}$$

XRD analysis of the extruded FILs and IND was performed using a Bruker D2 Phaser diffractometer (Bruker AXS Inc., Billerica, MA, USA) equipped with a CuKα radiation source (λ = 1.5418 Å). Diffraction patterns were recorded at an operating voltage of 30 kV and a current of 10 mA. Samples were scanned over a 2θ range of 5° to 45° in continuous scan mode, using a step size of 0.02° and a scanning rate of 2°/min, corresponding to a counting time of 1.2 s per step. The obtained diffractograms were analyzed to evaluate the crystalline or amorphous nature of IND within the FIL matrix.

### 4.6. FDM 3D Printing and Geometry Screening

3D-printed dosage forms were developed using the optimized IND-loaded FILs produced via HME. The objective was to systematically investigate the influence of geometric architecture on printing efficiency, mass accuracy, and electrical energy consumption during FDM.

Three distinct geometries were selected to represent different structural and morphological characteristics:

- Capsule-shaped geometry, characterized by an elongated structure and relatively high surface-area-to-volume ratio.
- Cylindrical tablet, selected as a conventional reference geometry widely used in pharmaceutical manufacturing.
- Torus geometry, incorporating an internal cavity and curvature transitions, representing a more complex structure with potential implications for internal stress distribution and material deposition dynamics.

All geometries were designed using Computer-Aided Design (CAD) software (version 2024). Digital models were exported in stereolithography (.stl) format. To ensure dose consistency, the dimensions of each geometry were adjusted such that the final printed dosage form contained 25 mg of IND.

FDM printing was performed using a Flashforge Creator 3 printer (Flashforge 3D Technology, Jinhua, China). During printing, electrical energy consumption was continuously monitored using a digital energy logger (Electrocorder AL-2VA), permanently connected to the printer power supply. To enable a controlled comparison among geometries, printing parameters were kept constant across all designs:

- Nozzle temperature: 150 °C;
- Build plate temperature: 35 °C;
- Printing speed: 10 mm/s.

Additional slicing parameters were identical for all geometries (Table 8):

### 4.7. DoE-Driven Optimization of the FDM 3D Printing Process

A full factorial 2^2^ DoE approach with a central point was employed to evaluate the influence of critical FDM process parameters on printing performance, electrical energy consumption, and drug content uniformity. Experiments were conducted using the cylindrical tablet geometry previously selected as optimal and the optimized IND-loaded FIL as feedstock.

Three independent variables were investigated: platform temperature (30–40 °C), nozzle temperature (150–160 °C), and printing speed (8–12 mm/s). These ranges were selected to ensure safe thermal processing of the ASDs while covering practically relevant FDM operational conditions. All other slicing parameters, including infill density, layer height, perimeter overlap, and infill pattern, were maintained constant throughout the study to isolate the effects of the selected factors.

Printing time (R_1_) was recorded directly from the printer software and verified experimentally. Electrical energy consumption (R_2_) was monitored using a digital energy logger (Electrocorder AL-2VA) connected to the printer power supply. IND encapsulation efficacy (R_3_) was determined by dissolving the printed dosage forms in dimethylformamide followed by UV-Vis spectrophotometric quantification at 318 nm using a validated calibration curve. Encapsulation efficiency was calculated according to Equation (4) as follows:

(4) $$Encapsulation\;efficiency\left(\%\right)=\frac{experimental\;IND\;concentration}{theoretical\;IND\;concentration}$$

Statistical analysis was performed at a 95% confidence level. Model adequacy was evaluated using ANOVA, regression diagnostics, and goodness-of-fit statistics. Response surface methodology was applied to interpret main effects and interactions among the investigated variables.

### 4.8. In Vitro Dissolution Studies of Optimized Dosage Forms

In vitro dissolution studies were performed to evaluate the drug release behavior of the optimized ASD-based 3D-printed tablets in comparison with IND. Dissolution testing was conducted in PBS, pH 6.8 using a total volume of 50 mL maintained at 37 ± 0.5 °C.

Samples were withdrawn at predetermined time intervals (5, 10, 15, 30, 45, 60, 90, 120, 180, 240, 300, 360, and 420 min) and analyzed for IND content using UV–Vis spectrophotometry at 318 nm. At each sampling point, an equivalent volume of fresh medium was added to maintain constant volume. All experiments were performed in triplicate (*n* = 3).

Non-sink conditions were intentionally maintained in all dissolution experiments to enable the development of supersaturation and better simulate the finite fluid volumes of the gastrointestinal (GI) tract. The degree of departure from sink conditions was quantified using the dimensionless sink index (SI), defined as:

$$SI=C_{s}*V/Dose$$

where C_s_ is the equilibrium solubility of crystalline IND, V is the volume of the dissolution medium, and Dose is the total amount of drug in the dosage form. In the present study, SI was approximately 0.4 for all experiments, confirming operation under non-sink conditions. According to established criteria, lower SI values (SI ≪ 3) indicate non-sink conditions, whereas higher values approach ideal sink conditions [44].

## Figures and Tables

**Table 1:** Experimental design matrix and corresponding response values obtained from the factorial DoE applied for the optimization of HME FILs.

Run	Factors			Responses		
	A (% *w*/*w*)	B (°C)	Y_1_ (KWh)	Y_2_ (%)	Y_3_ (%)	Y_4_ (%)
1	12.5	130.0	0.161	37.99	85.61	0.00
2	20.0	150.0	0.168	33.67	88.36	0.00
3	20.0	150.0	0.165	35.98	91.61	0.00
4	20.0	110.0	0.147	43.97	86.8	7.43
5	5.0	110.0	0.152	36.82	92.76	0.00
6	5.0	150.0	0.182	24.11	111.46	0.00
7	20.0	110.0	0.149	43.91	86.68	7.48
8	12.5	130.0	0.163	41.59	85.55	0.00
9	20.0	110.0	0.144	47.12	86.71	7.46
10	5.0	110.0	0.154	37.71	93.31	0.00
11	12.5	130.0	0.164	40.42	85.17	0.00
12	5.0	150.0	0.184	27.32	111.97	0.00
13	5.0	150.0	0.188	24.17	111.61	0.00
14	20.0	150.0	0.163	32.35	95.17	0.00
15	5.0	110.0	0.156	40.36	93.57	0.00

**Table 2:** ANOVA results for the DoE applied to HME processing at a significance level of α = 0.05. All non-significant factors and factors’ interactions were excluded by the table for clarity.

Factors	Y_1_		Y_2_		Y_3_		Y_4_	
	F-Value	*p*-Value	F-Value	*p*-Value	F-Value	*p*-Value	F-Value	*p*-Value
A	59.54	<0.0001	26.06	0.0003	20.40	0.009	-	-
B	213.73	0.0003	62.95	<0.0001	16.03	0.0021	-	-
AB	10.55	<0.0001	-	-	5.30	0.0419	-	-

**Table 3:** Statistical diagnostics tools: R^2^, adjusted R^2^, predicted R^2^, and adequate precision for the HME experimental design.

Response	R^2^	Adj. R^2^	Pred. R^2^	Adeq. Precision
Y_1_	0.9627	0.9525	0.9221	24.97
Y_2_	0.8812	0.8614	0.8323	16.83
Y_3_	0.7914	0.7345	0.7052	9.53
Y_4_	-	-	-	-

**Table 4:** Comparative evaluation of electrical energy demand, printing duration, weight yield, and FIL usage during FDM 3D printing of dosage forms with distinct geometrical architectures. Values correspond to single-run measurements obtained under identical processing conditions.

Dosage Form	Energy Demand (kWh)	Energy Demand/Printed Mass (kWh/g)	Printing Time	Weight Yield	FIL Required for Printing
Capsule	0.1498	0.297	20 min 20 s	0.84	0.6 g/0.23 m
Tablet	0.1498	0.283	18 min 25 s	0.88	0.6 g/0.23 m
Torus	0.1608	0.344	21 min 30 s	0.78	0.6 g/0.23 m

**Table 5:** Experimental design matrix and corresponding response values obtained from the full factorial DoE applied for the optimization of FDM 3D-printing process.

Run	Factors			Responses		
	X_1_ (°C)	X_2_ (°C)	X_3_ (mm/s)	R_1_ (h)	R_2_ (kWh)	R_3_ (%)
1	40	150	12	0.238	0.044	96.40
2	30	150	12	0.211	0.028	85.20
3	30	160	8	0.227	0.031	95.20
4	40	150	12	0.233	0.044	95.90
5	40	150	8	0.240	0.045	92.70
6	40	160	8	0.245	0.047	92.40
7	30	150	12	0.212	0.026	85.20
8	35	155	10	0.230	0.038	94.60
9	40	160	8	0.246	0.048	92.50
10	30	150	8	0.220	0.028	89.70
11	40	150	8	0.241	0.047	92.00
12	30	150	8	0.234	0.030	90.20
13	40	160	12	0.218	0.047	93.84
14	40	160	12	0.229	0.046	92.70
15	40	160	12	0.224	0.046	93.73
16	40	150	12	0.241	0.043	96.00
17	40	150	8	0.235	0.047	91.00
18	30	150	8	0.236	0.029	91.30
19	30	160	12	0.191	0.029	93.98
20	30	160	12	0.214	0.030	93.95
21	30	160	8	0.224	0.031	96.20
22	30	160	8	0.204	0.032	96.24
23	30	160	12	0.211	0.029	95.00
24	30	150	12	0.194	0.027	85.20
25	40	160	8	0.232	0.048	92.00

**Table 6:** ANOVA results for the DoE applied to FDM processing at a significance level of α = 0.05. All non-significant factors and factors’ interactions were excluded by the table for clarity.

Factors	R_1_		R_2_		R_3_	
	F-Value	*p*-Value	F-Value	*p*-Value	F-Value	*p*-Value
X_1_	28.92	0.0003	2560.97	<0.0001	41.12	<0.0001
X_2_	2.45	<0.0001	41.53	<0.0001	99.07	<0.0001
X_3_	13.81	-	38.64	<0.0001	1.36	-
X_1_X_2_	0.00	0.0012	0.44	-	185.74	<0.0001
X_1_X_3_	1.48	-	0.00	-	95.57	<0.0001
X_2_X_3_	0.10	-	0.68	-	0.20	-

**Table 7:** Summary of statistical diagnostic parameters (R^2^, adjusted R^2^, predicted R^2^, and adequate precision) for the FDM 3D printing DoE models.

Response	R^2^	Adj. R^2^	Pred. R^2^	Adeq. Precision
Y_1_	0.7220	0.6294	0.5824	8.23
Y_2_	0.9936	0.9910	0.9858	44.98
Y_3_	0.9638	0.9489	0.9373	25.43

**Table 8:** Operational FDM printing parameters applied during fabrication of the investigated dosage form geometries.

Parameter	Value
Fill density	40%
Fill pattern	Line
Layer height	0.18 mm
First layer height	0.27 mm
Start angle	45°
Overlap perimeter	40%
Base Print Speed	10 mm/s
Travel speed	15 mm/s
First layer maximum speed	5 mm/s
First layer maximum travel speed	5 mm/s

## Data Availability

The original contributions presented in this study are included in the article/Supplementary Materials. Further inquiries can be directed to the corresponding author.

## Supplementary Materials

The following supporting information can be downloaded at: https://www.mdpi.com/article/10.3390/ph19040562/s1, Figure S1: Average voltage profiles recorded during hot-melt extrusion for each DoE condition (mean of triplicate runs). The profiles are grouped as follows: (**a**) Runs 1, 8, and 11; (**b**) Runs 2, 3, and 14; (**c**) Runs 4, 7, and 9; (**d**) Runs 5, 10, and 15; and (**e**) Runs 6, 12, and 13. Each group corresponds to identical formulation–temperature conditions as defined in the factorial design; Figure S2: Average current profiles recorded during hot-melt extrusion for each DoE condition (mean of triplicate runs). The profiles are grouped as follows: (**a**) Runs 1, 8, and 11; (**b**) Runs 2, 3, and 14; (**c**) Runs 4, 7, and 9; (**d**) Runs 5, 10, and 15; and (**e**) Runs 6, 12, and 13. Each group corresponds to identical formulation–temperature conditions as defined in the factorial design; Figure S3: Statistical diagnostic plots for HME DoE: (**a**) normal probability plot of residuals, (**b**) residuals versus predicted values, (**c**) residuals versus run order, and (**d**) Cook’s distance; Figure S4: (**a**) Average voltage and (**b**) current intensity profiles recorded during hot-melt extrusion of the optimized filament; Figure S5: Representative images of the 3D-printed dosage forms fabricated from the optimized filament, illustrating the three investigated geometries: (**a**) capsule, (**b**) cylindrical tablet, and (**c**) torus; Figure S6: Recorded voltage profiles during FDM 3D printing of the (**a**) capsule, (**b**) cylindrical tablet, and (**c**) torus geometries; Figure S7: Recorded current intensity profiles during FDM 3D printing of the (**a**) capsule, (**b**) cylindrical tablet, and (**c**) torus geometries; Figure S8: Average voltage profiles recorded during FDM 3D printing for each DoE condition (mean of triplicate runs). The profiles are grouped according to the investigated factors: (Χ_1_) platform temperature, (Χ_2_) nozzle temperature, and (Χ_3_) printing speed; Figure S9: Average current intensity profiles recorded during FDM 3D printing for each DoE condition (mean of triplicate runs). The profiles are grouped according to the investigated factors: (X_1_) platform temperature, (X_2_) nozzle temperature, and (X_3_) printing speed; Figure S10: Statistical diagnostic plots for FDM 3D printing DoE: (**a**) normal probability plot of residuals, (**b**) residuals versus predicted values, (**c**) residuals versus run order, and (**d**) Cook’s distance; Figure S11: Voltage and current intensity profiles recorded during FDM 3D printing under the validated optimal processing conditions (platform temperature 32 °C, nozzle temperature 158 °C, printing speed 11 mm/s).

## Abbreviations

The following abbreviations are used in this manuscript:

ANOVA	Analysis of variance
API	Active pharmaceutical ingredient
ASD	Amorphous solid dispersion
CA	Citric acid
DSC	Differential scanning calorimetry
DoE	Design of experiments
FDM	Fused deposition modeling
FIL	Filament
GI	Gastrointestinal
HME	Hot-melt extrusion
HSM	Hot-stage microscopy
IND	Indomethacin
MAN	Mannitol
PBS	Phosphate buffer solution
PEG	Polyethylene glycol
PM	Physical mixture
PVA	Polyvinyl alcohol
R^2^	Coefficient of determination
SOL	Soluplus
TEC	Triethyl citrate
Tg	Glass transition temperature
TGA	Thermogravimetric analysis
XRD	X-ray diffraction
