# Supplementary material for: Sustainable Design and DoE-Based Optimization of Polymeric Systems for FDM 3D-Printed Indomethacin Amorphous Solid Dispersions
Source: Pharmaceuticals (Basel). 2026 Apr 1;19(4):562. doi: 10.3390/ph19040562 (PMC13118288; doi:10.3390/ph19040562)
Supplement: Supplementary file 1 [file pharmaceuticals-19-00562-s001.zip › pharmaceuticals-4211235-SI.pdf]

Supplementary Information

Sustainable Design and DoE-Based Optimization of Polymeric Systems for FDM 3D-Printed Indomethacin Amorphous Solid Dispersions

Ioannis Pantazos [1], Christos Cholevas[1], Christos Vlachokostas[2] Afroditi Kapourani[1,*], and Panagiotis Barmpalexis [1,3].

[1] Laboratory of Pharmaceutical Technology, Division of Pharmaceutical Technology, School of Pharmacy, Faculty of Health Sciences, Aristotle University of Thessaloniki, 541 24 Thessaloniki, Greece.

[2] Sustainability Engineering Laboratory, Department of Mechanical Engineering, Aristotle University of Thessaloniki, 541 24 Thessaloniki, Greece.

[3] Natural Products Research Centre of Excellence-AUTH (NatPro-AUTH), Center for Interdisciplinary Research and Innovation (CIRI-AUTH), Thessaloniki 57001, Greece.

Corresponding author information

*Afroditi Kapourani, PhD

Department of Pharmaceutical Technology,

School of Pharmacy, Aristotle University of Thessaloniki

Thessaloniki 54124 (Greece)

Email: akapourag@pharm.auth.gr

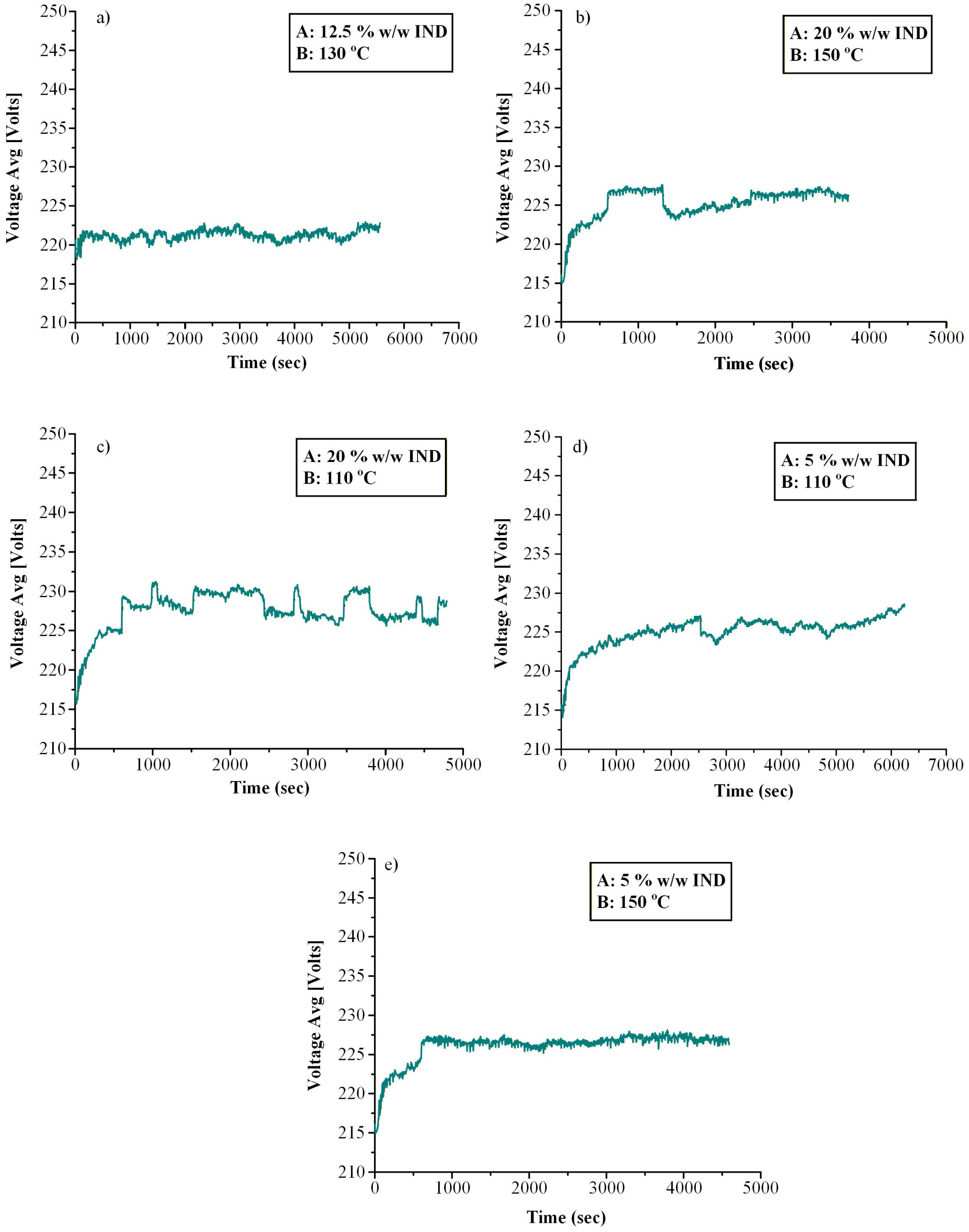

Figure S1. Average voltage profiles recorded during hot-melt extrusion for each DoE condition (mean of triplicate runs). The profiles are grouped as follows: **a**) Runs 1, 8, and 11; **b**) Runs 2, 3, and 14; **c**) Runs 4, 7, and 9; **d**) Runs 5, 10, and 15; and **e**) Runs 6, 12, and 13. Each group corresponds to identical formulation-temperature conditions as defined in the factorial design.

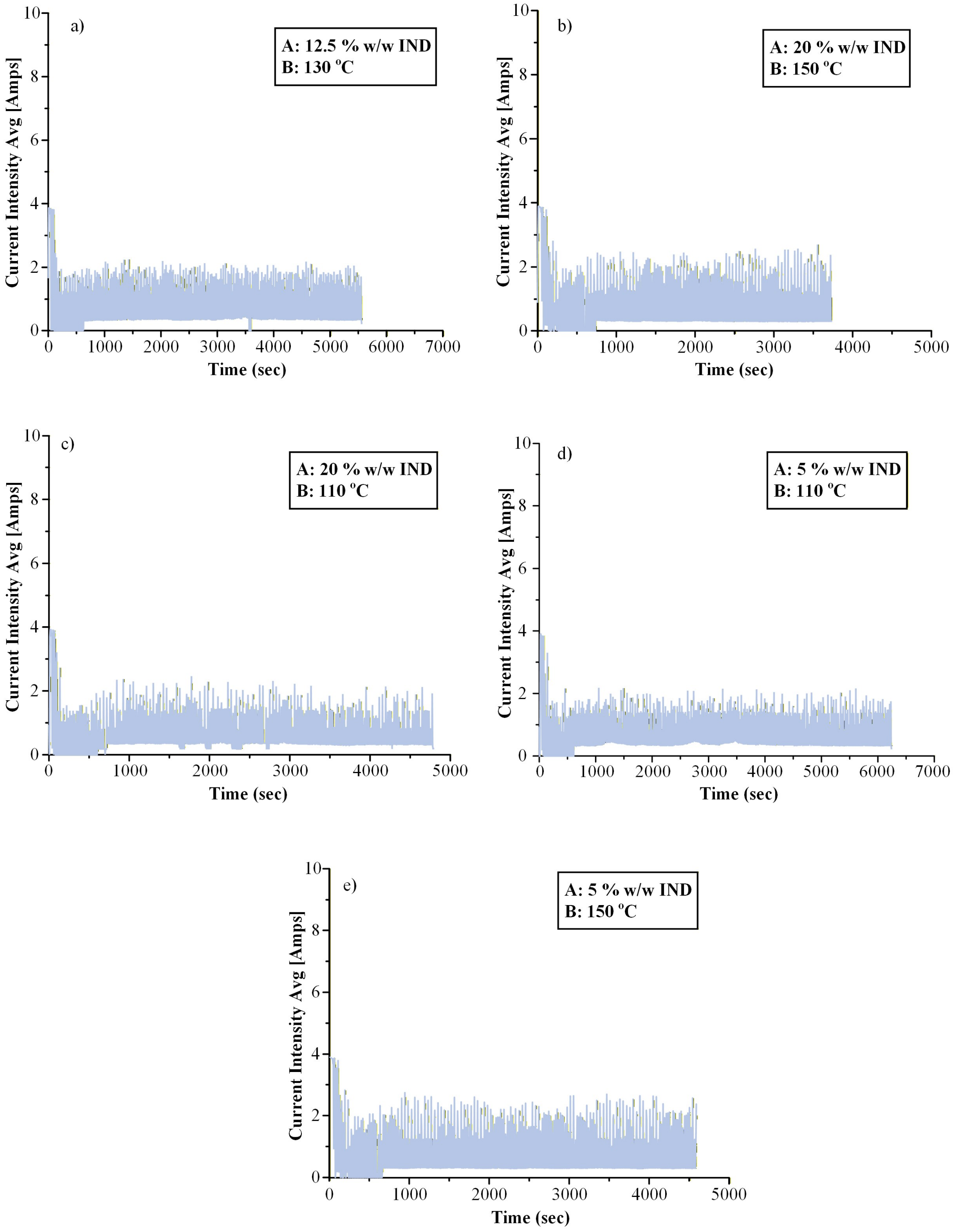

Figure S2. Average current profiles recorded during hot-melt extrusion for each DoE condition (mean of triplicate runs). The profiles are grouped as follows: **a**) Runs 1, 8, and 11; **b**) Runs 2, 3, and 14; **c**) Runs 4, 7, and 9; **d**) Runs 5, 10, and 15; and **e**) Runs 6, 12, and 13. Each group corresponds to identical formulation-temperature conditions as defined in the factorial design.

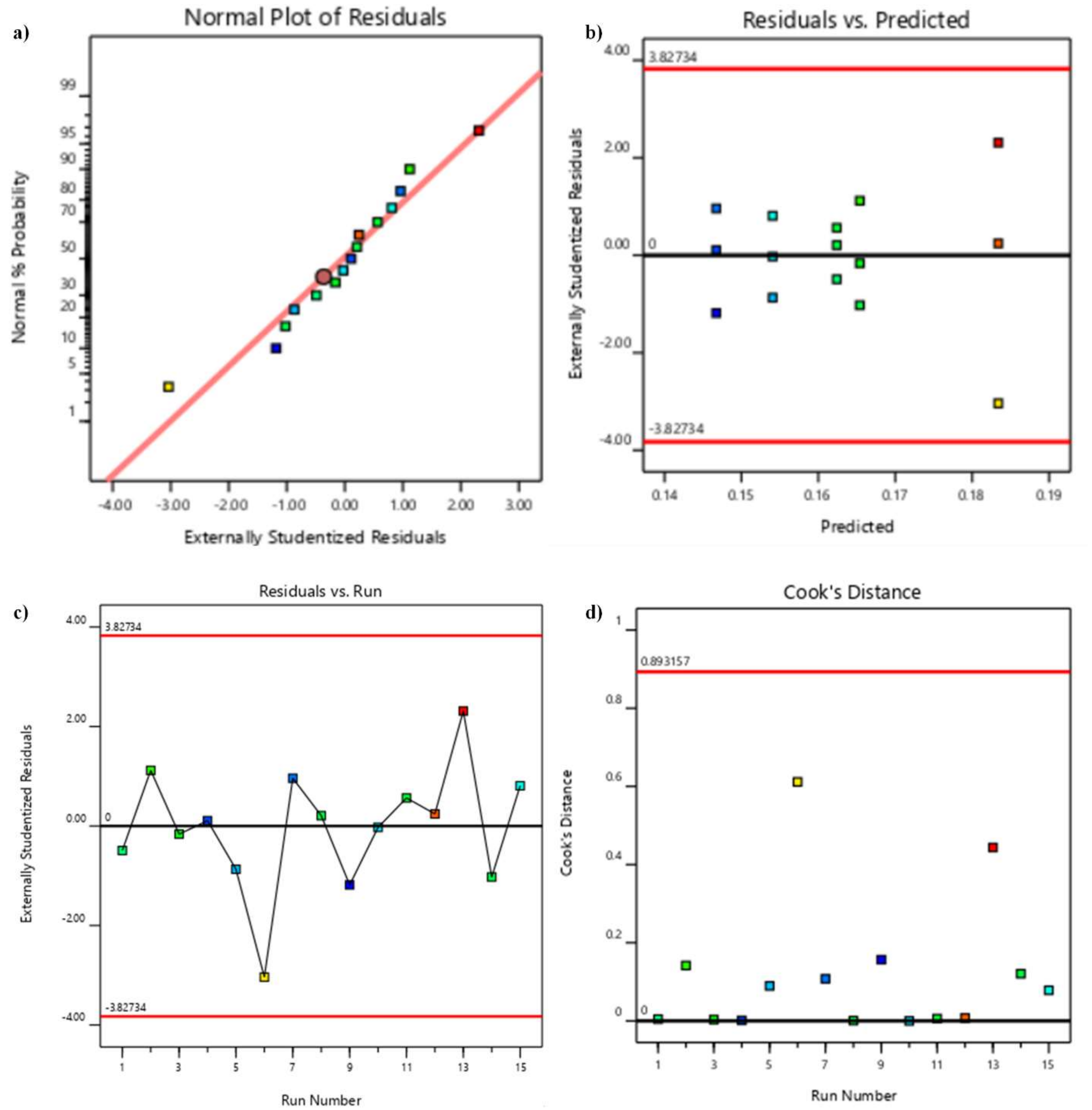

Figure S3. Statistical diagnostic plots for HME DoE: **a**) normal probability plot of residuals, **b**) residuals versus predicted values, **c**) residuals versus run order, and **d)** Cook's distance.

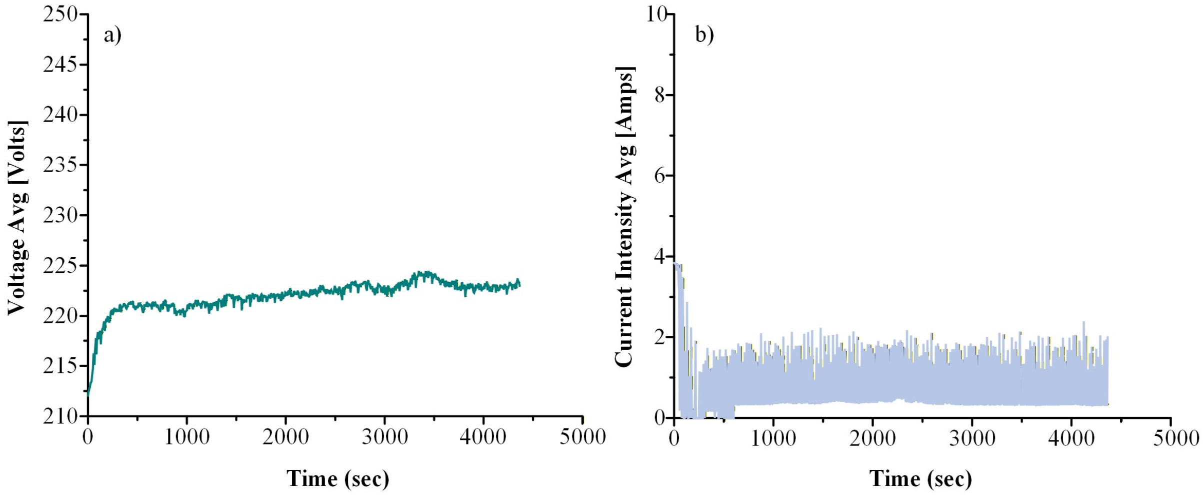

Figure S4. a) Average voltage and **b**) current intensity profiles recorded during hot-melt extrusion of the optimized filament.

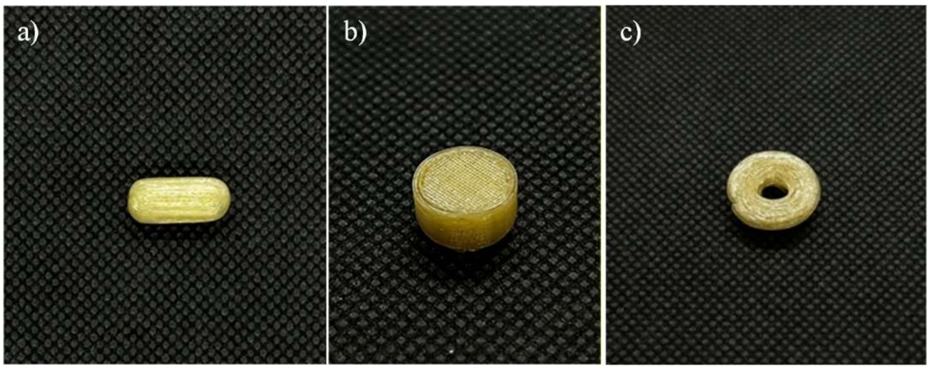

Figure S5. Representative images of the 3D-printed dosage forms fabricated from the optimized filament, illustrating the three investigated geometries: **a**) capsule, **b**) cylindrical tablet, and **c**) torus.

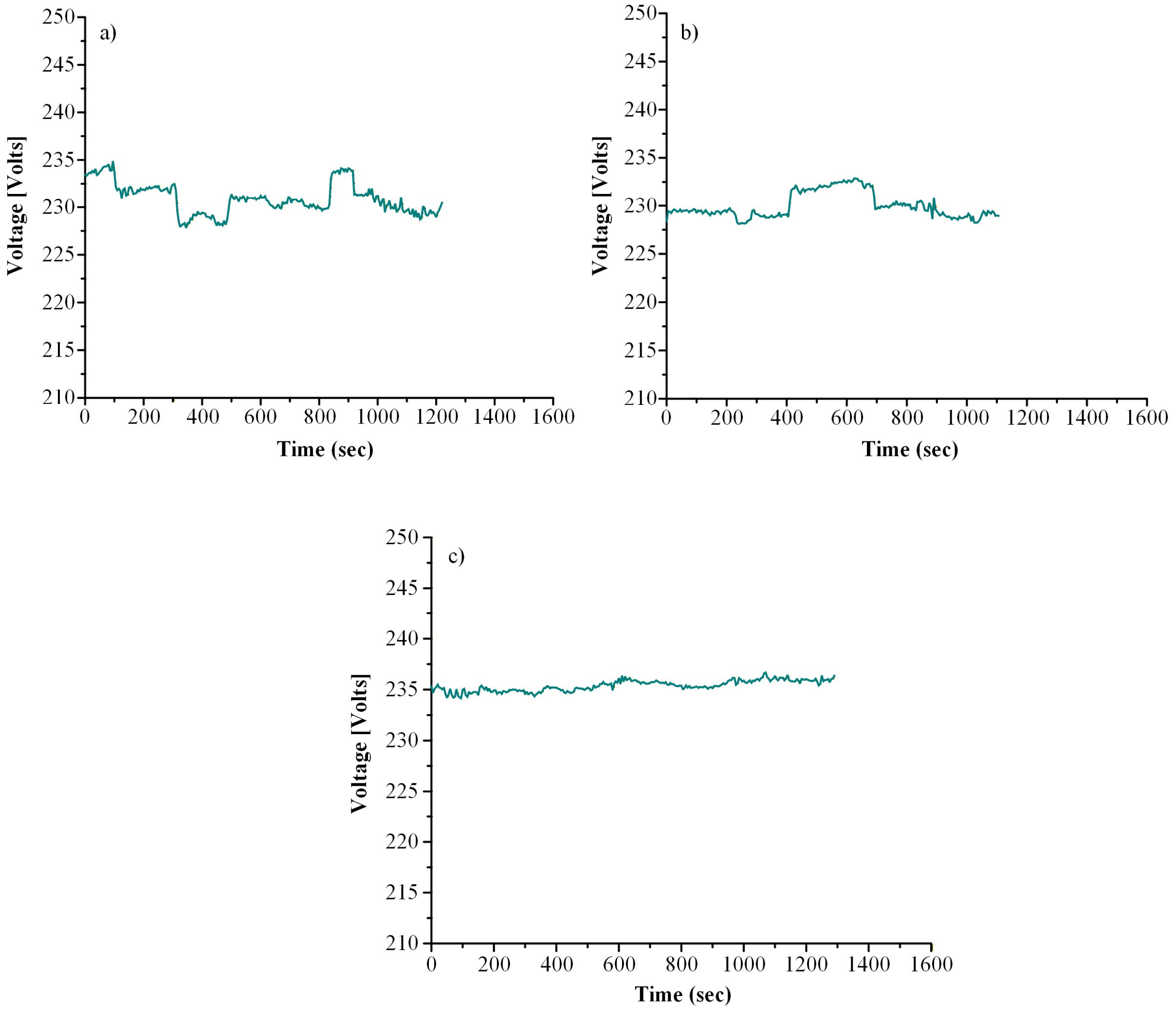

Figure S6. Recorded voltage profiles during FDM 3D printing of the **a**) capsule, **b**) cylindrical tablet, and **c**) torus geometries.

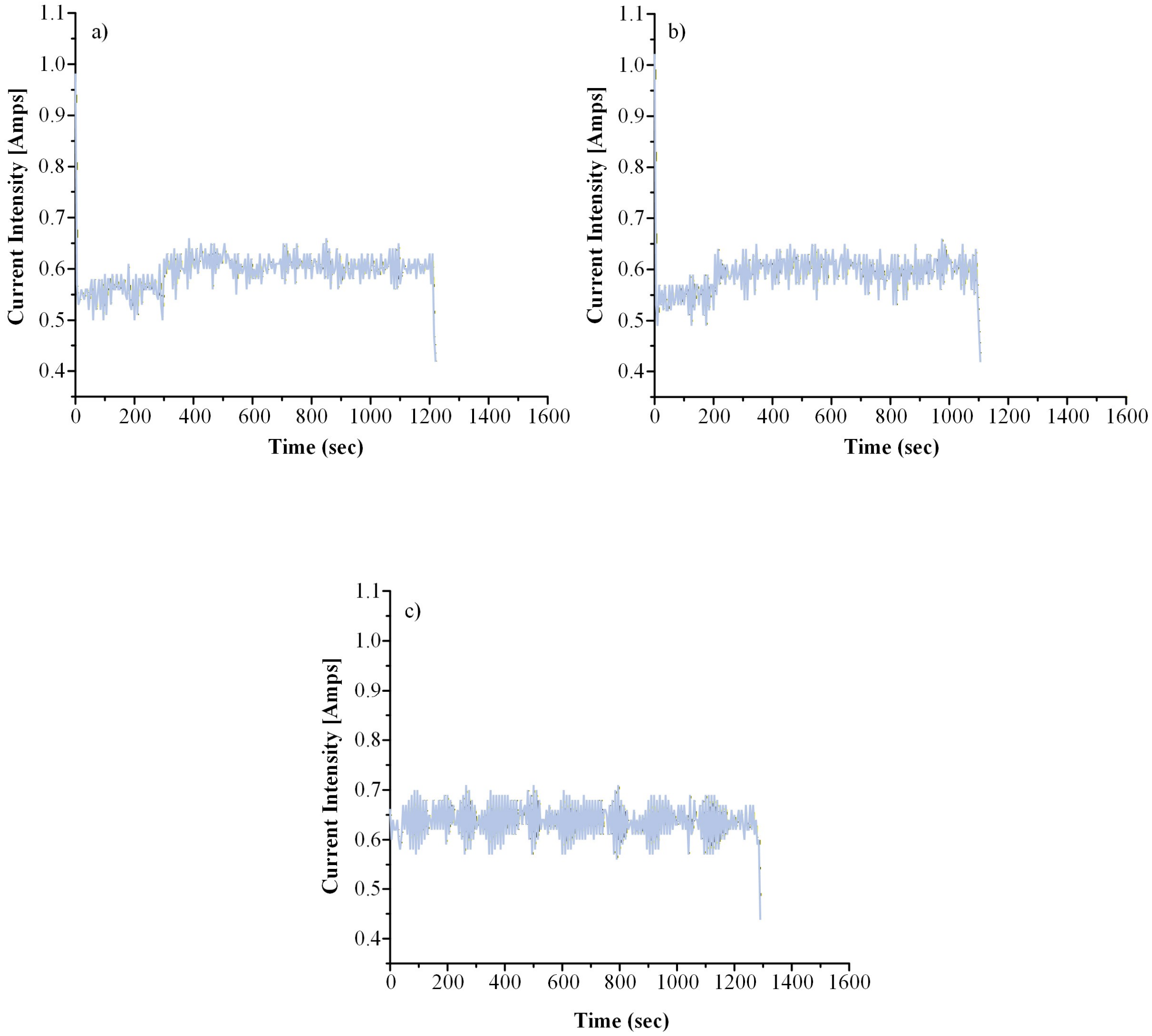

Figure S7. Recorded current intensity profiles during FDM 3D printing of the **a**) capsule, **b**) cylindrical tablet, and **c**) torus geometries.

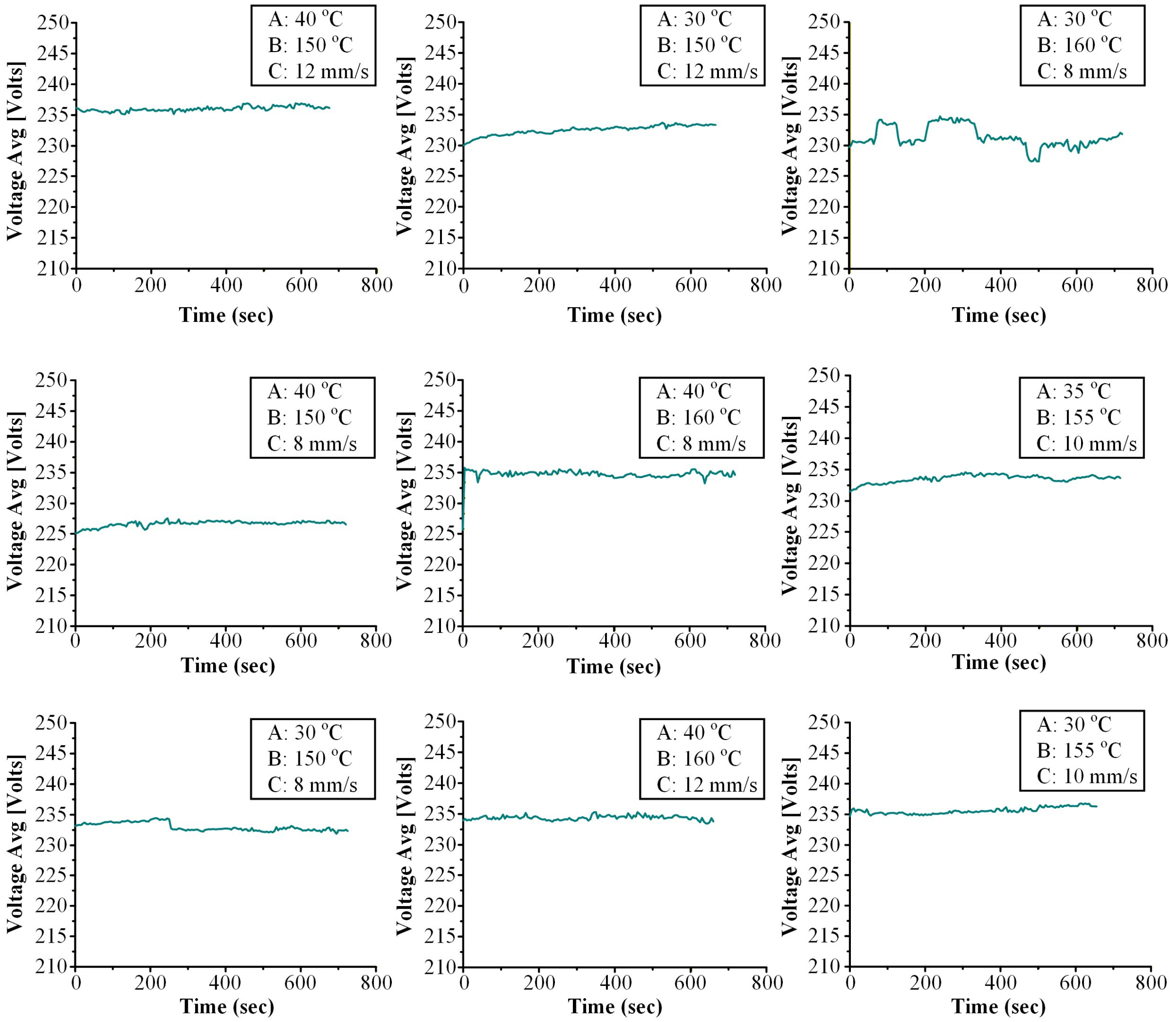

Figure S8. Average voltage profiles recorded during FDM 3D printing for each DoE condition (mean of triplicate runs). The profiles are grouped according to the investigated factors: (X_1) platform temperature, (X_2) nozzle temperature, and (X_3) printing speed.

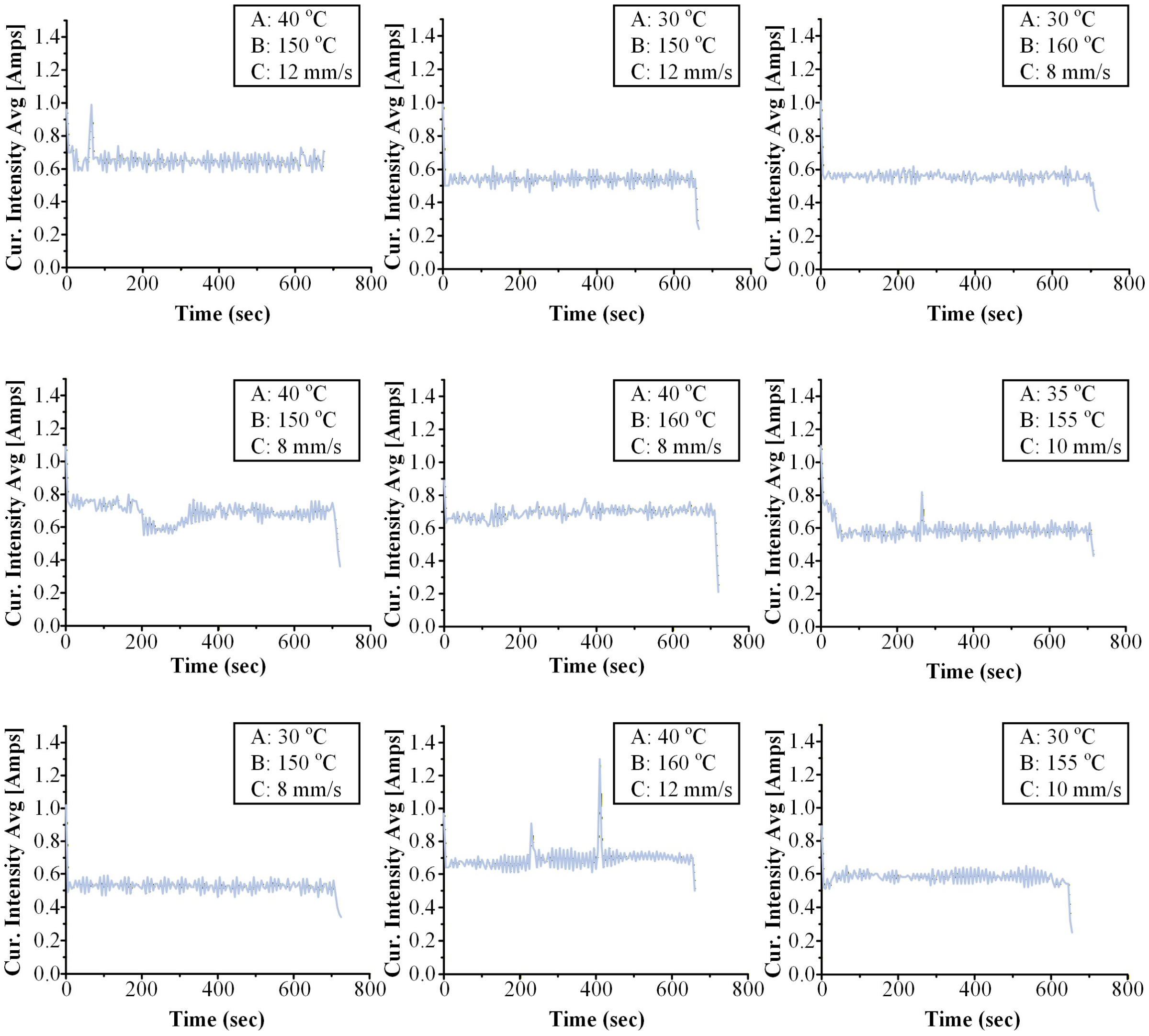

Figure S9. Average current intensity profiles recorded during FDM 3D printing for each DoE condition (mean of triplicate runs). The profiles are grouped according to the investigated factors: (X_1) platform temperature, (X_2) nozzle temperature, and (X_3) printing speed.

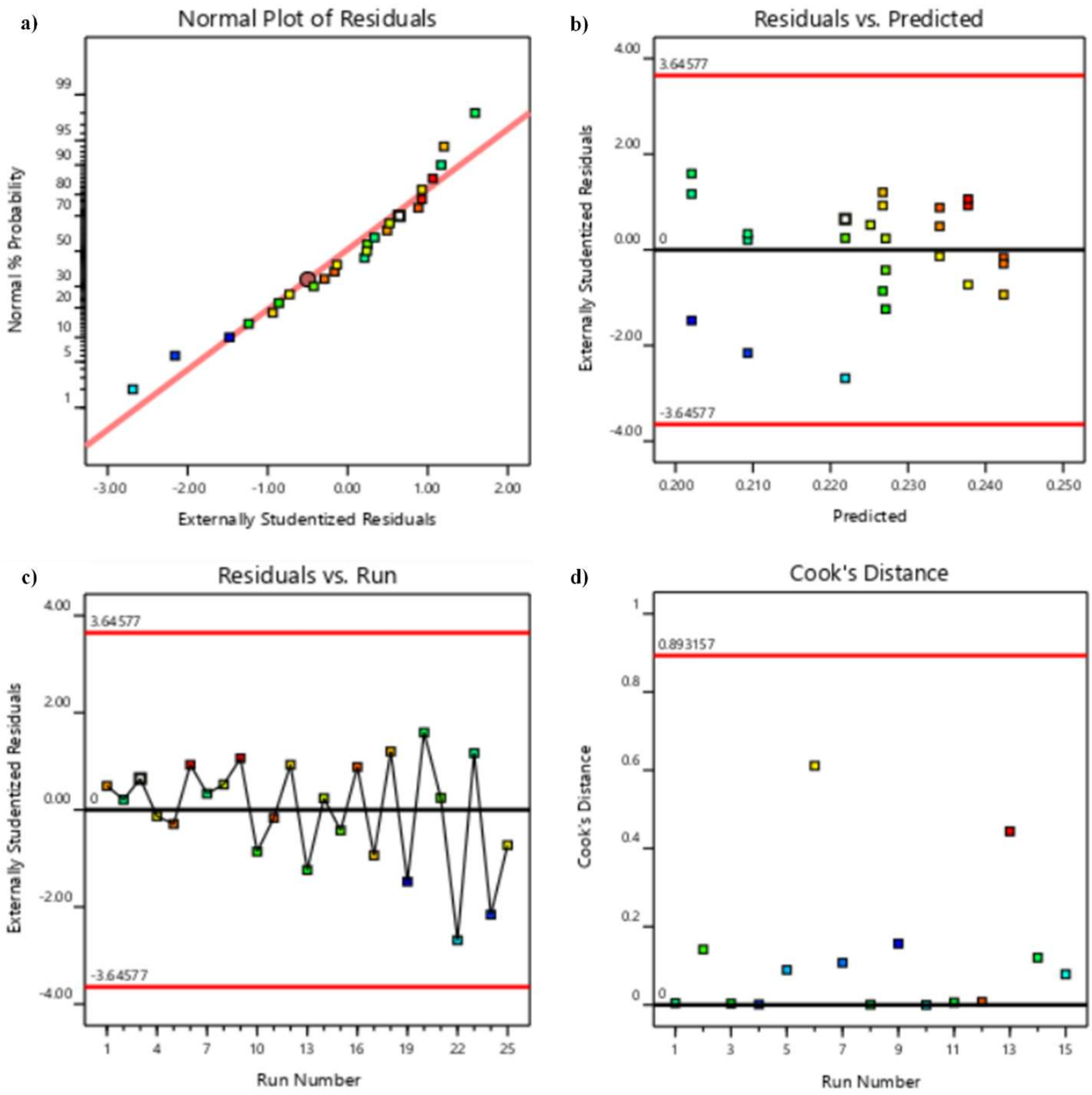

Figure S10. Statistical diagnostic plots for FDM 3D printing DoE: **a**) normal probability plot of residuals, **b**) residuals versus predicted values, **c**) residuals versus run order, and **d**) Cook’s distance.

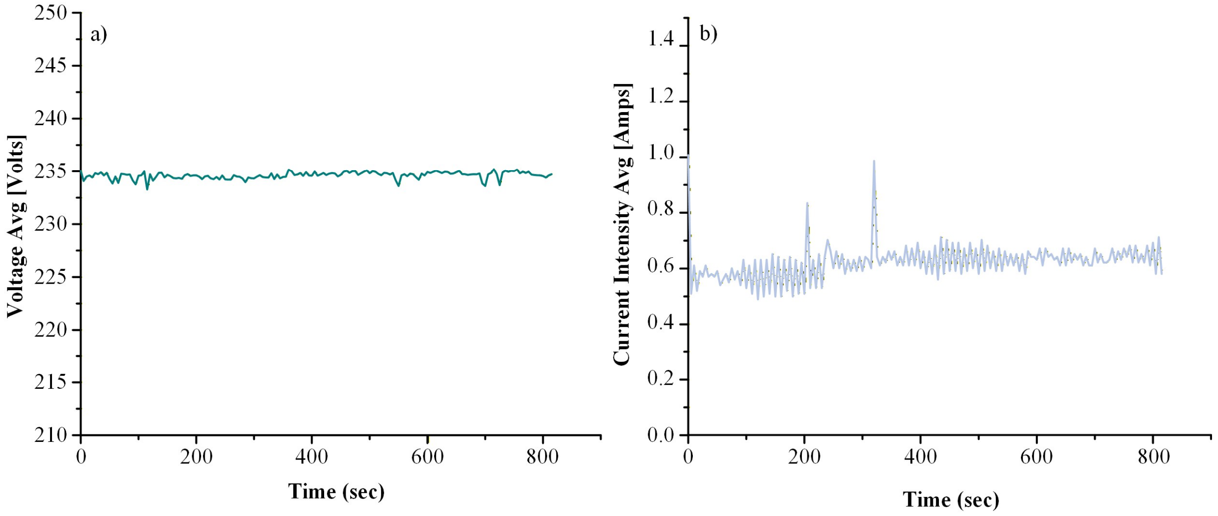

Figure S11. Voltage and current intensity profiles recorded during FDM 3D printing under the validated optimal processing conditions (platform temperature 32 °C, nozzle temperature 158 °C, printing speed 11 mm/s).
